# Supplementary material for: Novel Thiochromanone Derivatives Containing a Sulfonyl Hydrazone Moiety: Design, Synthesis, and Bioactivity Evaluation
Source: Molecules. 2021 May 14;26(10):2925. doi: 10.3390/molecules26102925 (PMC8156870; doi:10.3390/molecules26102925)
Supplement: Supplementary file 1 [file molecules-26-02925-s001.zip › molecules-1208152-supplementary.pdf]

Novel Thiochromanone Derivatives Containing a Sulfonyl Hydrazone Moiety: Design, Synthesis, and Bioactivity Evaluation

Lu Yu [1], Jiyan Chi [1], Lingling Xiao [1], Jie Li [1], Zhangfei Tang [1], Shuming Tan [1,*] and Pei Li [1,2,*]

[1] School of Liquor and Food Engineering, Guizhou University, Guiyang 550025, China; lyu1@gzu.edu.cn (L.Y.); qq1401064120@126.com (J.C.); an1378386891@163.com (L.X.); sushilee0120@163.com (J.L.); tzf18885247153@163.com (Z.T.)

[2] Qiandongnan Engineering and Technology Research Center for Comprehensive Utilization of National Medicine, Kaili University, Kaili 556011, China

* Correspondence: smtan@gzu.edu.cn (S.T.); pl19890627@126.com or lipei@kluniv.edu.cn (P.L.); Tel.: +86-(0851)8559466

Data for methyl (Z)-6-fluoro-4-(2-(phenylsulfonyl)hydrazineylidene)thiochromane-2-carboxylate (**4a**). White solid; mp 175–176 °C; yield 65.7%; ^{1}H NMR (400 MHz, DMSO-*d*$_6$, ppm) δ: 10.94 (s, 1H, NH), 7.97-7.95 (m, 2H, Ph-H), 7.47 (dd, J1 = 3.2 Hz, J2 = 10.4 Hz, 1H, Ph-H), 7.30 (dd, J1 = 5.6 Hz, J2 = 8.8 Hz, 1H, Ph-H), 7.19-7.14 (m, 1H, Ph-H), 4.39 (q, J = 1.2 Hz, 1H, SCH), 3.62 (s, 3H, CH_3), 3.17-3.04 (m, 2H, CH_2); ^{13}C NMR (100 MHz, DMSO-*d*$_6$, ppm) δ: 170.7, 161.8, 159.4, 148.8 (d, J = 3.0 Hz), 139.3, 133.7, 130.6, 130.5, 129.6, 128.6, 128.1, 118.0, 117.7, 112.1, 111.9, 53.1, 40.6, 30.2; HRMS (ESI) [M+Na]$^+$ calcd for $C_{17}H_{15}N_2O_4S_2$: 417.03494, found: 417.03466.

Data for methyl (Z)-6-fluoro-4-(2-tosylhydrazineylidene)thiochromane-2-carboxylate (**4b**). White solid; mp 178–179 °C; yield 72.1%; ^{1}H NMR (400 MHz, DMSO-*d*$_6$, ppm) δ: 10.83 (s, 1H, NH), 7.83 (d, J = 8.4 Hz, 2H, Ph-H), 7.49-7.43 (m, 3H, Ph-H), 7.30 (q, J = 4.2 Hz, 1H, Ph-H), 7.19-7.14 (m, 1H, Ph-H), 4.38 (q, J = 4.8 Hz, 1H, SCH), 3.62 (s, 3H, CH_3), 3.14-3.02 (m, 2H, CH_2); ^{13}C NMR (100 MHz, DMSO-*d*$_6$, ppm) δ: 170.7, 161.9, 159.5, 148.5 (d, J = 2.0 Hz), 144.1, 136.4, 130.5, 130.0, 128.1, 117.9, 117.6, 112.1, 111.9, 53.1, 40.2, 30.3, 21.5; HRMS (ESI) [M+Na]$^+$ calcd for $C_{18}H_{17}N_2O_4S_2$: 431.05060, found: 431.05055.

Data for methyl (Z)-6-fluoro-4-(2-((4-fluorophenyl)sulfonyl)hydrazineylidene)thiochromane-2-carboxylate (**4c**). White solid; mp 170–171 °C; yield 74.2%; ^{1}H NMR (400 MHz, DMSO-*d*$_6$, ppm) δ: 10.96 (s, 1H, NH), 8.05-8.00 (m, 2H, Ph-H), 7.53-7.48 (m, 3H, Ph-H), 7.31 (dd, J1 = 5.6 Hz, J2 = 8.8 Hz, 1H, Ph-H), 7.21-7.16 (m, 1H, Ph-H), 4.40 (q, J1 = 4.8 Hz, J2 = 6.8 Hz, 1H, SCH), 3.63 (s, 3H, CH_3), 3.18-3.05 (m, 2H, CH_2); ^{13}C NMR (100 MHz, DMSO-*d*$_6$, ppm) δ: 170.7, 166.4, 163.9, 161.9, 159.5, 149.2, 135.5 (d, J = 3.0 Hz), 132.8 (d, J = 7.0 Hz), 131.2 (d, J = 10.0 Hz), 130.6 (d, J = 8.0 Hz), 128.6, 118.0, 117.8, 116.8, 112.1, 111.9, 53.1, 40.4, 30.3; HRMS (ESI) [M+Na]$^+$ calcd for $C_{17}H_{15}F_2N_2O_4S_2$: 435.02553, found: 435.02509.

Data for ethyl (Z)-6-fluoro-4-(2-(phenylsulfonyl)hydrazineylidene)thiochromane-2-carboxylate (**4d**). White solid; mp 167–169 °C; yield 84.6%; ^{1}H NMR (400 MHz, DMSO-*d*$_6$, ppm) δ: 10.93 (s, 1H, NH), 7.97-7.95 (m, 2H, Ph-H), 7.71-7.62 (m, 3H, Ph-H), 7.46 (dd, J1 = 2.8 Hz, J2 = 10.8 Hz, 1H, Ph-H), 7.30 (dd, J1 = 5.6 Hz, J2 = 8.8 Hz, 1H, Ph-H), 7.19-7.14 (m, 1H, Ph-H), 4.36 (q, J1 = 4.4 Hz, J2 = 6.4 Hz, 1H, SCH), 4.10-4.02 (m, 2H, CH_2), 3.18-3.02 (m, 2H, CH_2), 1.09 (q, J1 = 6.8 Hz, J2 = 14.0 Hz, 3H, CH_3); ^{13}C NMR (100 MHz, DMSO-*d*$_6$, ppm) δ: 170.1, 161.8, 159.4, 148.9 (d, J = 3.0 Hz), 139.3, 133.7, 130.6, 130.5, 129.6, 128.6, 128.1, 117.9, 117.7, 112.1, 111.9, 61.9, 40.4, 30.3, 14.3; HRMS (ESI) [M+Na]$^+$ calcd for $C_{18}H_{17}FN_2O_4S_2$: 431.05060, found: 431.05044.

Data for ethyl (Z)-6-fluoro-4-(2-tosylhydrazineylidene)thiochromane-2-carboxylate (**4e**). White solid; mp 159–161 °C; yield 68.7%; ^{1}H NMR (400 MHz, DMSO-*d*$_6$, ppm) δ: 10.83 (s, 1H, NH), 7.83 (d, J = 8.4 Hz, 2H, Ph-H), 7.48-7.43 (m, 3H, Ph-H), 7.29 (dd, J1 = 5.6 Hz, J2 = 8.8 Hz, 1H, Ph-H), 7.19-7.14 (m, 1H, Ph-H), 4.35 (q, J1 = 4.4 Hz, J2 = 6.4 Hz, 1H, SCH), 4.09-4.03 (m, 2H, $\underline{CH_2}CH_3$), 3,15-3.00 (m, 2H, CH_2), 2.51 (q, J1 = 1.6 Hz, J2 = 4.0 Hz, 3H, $CH_2\underline{CH_3}$), 2.38 (s, 3H, CH_3); ^{13}C NMR (100 MHz, DMSO-*d*$_6$, ppm) δ: 170.1, 162.1, 159.4, 144.2, 136.4, 133.1, 130.6, 130.5, 130.1, 128.6, 128.1, 117.9, 117.6, 112.1, 111.8, 61.9, 40.4, 30.3, 21.5, 14.3; HRMS (ESI) [M+Na]$^+$ calcd for $C_{19}H_{19}FN_2O_4S_2$: 445.06625, found: 445.06647.

Data for ethyl (Z)-6-fluoro-4-(2-((4-fluorophenyl)sulfonyl)hydrazineylidene)thiochromane-2-carboxylate (**4f**). White solid; mp 152–154 °C; yield 73.2%; ^{1}H NMR (400 MHz, DMSO-*d*$_6$, ppm) δ: 10.95 (s, 1H, NH), 8.05-8.01 (m, 2H, Ph-H), 7.53-7.47 (m, 3H, Ph-H), 7.31 (dd, J1 = 5.6 Hz, J2 = 8.8 Hz, 1H, Ph-H), 7.20-7.15 (m, 1H, Ph-H), 4.37 (q, J1 = 4.8 Hz, J2 = 6.8 Hz, 1H, SCH), 4.11-4.05 (m, 2H, $\underline{CH_2}CH_3$), 3.19-3.03 (m, 2H, CH_2), 1.10 (t, J = 7.6 Hz, 3H, $CH_2\underline{CH_3}$); ^{13}C NMR (100 MHz, DMSO-*d*$_6$, ppm) δ: 170.1, 166.4, 163.9, 161.9, 159.4, 149.3, 135.5 (d, J = 3.0 Hz), 132.9, 131.2 (d, J = 10 Hz), 130.6, 130.5, 128.7, 118.0, 117.7, 117.0, 116.8, 112.1, 111.9, 61.9, 40.6, 30.3, 14.3; HRMS (ESI) [M+Na]$^+$ calcd for $C_{18}H_{17}F_2N_2O_4S_2$: 449.04118, found: 449.04110.

Data for methyl (Z)-6-chloro-4-(2-(phenylsulfonyl)hydrazineylidene)thiochromane-2-carboxylate (**4g**). White solid; mp 192–194 °C; yield 71.0%; ^{1}H NMR (400 MHz, DMSO-*d*$_6$, ppm) δ: 10.98 (s, 1H, NH),

7.96-7.94 (m, 2H, Ph-H), 7.72-7.63 (m, 4H, Ph-H), 7.34-7.27 (m, 2H, Ph-H), 4.42 (q, J = 4.2 Hz, 1H, SCH), 3.62 (s, 3H, CH_3), 3.19-3.04 (m, 2H, CH_2); ^{13}C NMR (100 MHz, DMSO-d_6, ppm) δ: 170.6, 148.4, 139.3, 133.8, 132.6, 132.0, 130.8, 130.3, 129.9, 129.6, 128.0, 125.3, 53.2, 40.2, 30.0; HRMS (ESI) $[M+Na]^+$ calcd for $C_{17}H_{15}ClN_2O_4S_2$: 433.00540, found: 433.00526.

Data for methyl (*Z*)-6-chloro-4-(2-tosylhydrazineylidene)thiochromane-2-carboxylate (**4h**). White solid; mp 169–170 °C; yield 70.2%; ^{1}H NMR (400 MHz, DMSO-d_6, ppm) δ: 10.88 (s, 1H, NH), 7.82 (d, J = 8.0 Hz, 2H, Ph-H), 7.71 (d, J = 2.4 Hz, 1H, Ph-H), 7.43 (d, J = 8.0 Hz, 2H, Ph-H), 7.34-7.26 (m, 2H, Ph-H), 4.41 (q, J = 4.8 Hz, 1H, SCH), 3.62 (s, 3H, CH_3), 3.17-3.02 (m, 2H, CH_2), 2.38 (s, 3H, CH_3); ^{13}C NMR (100 MHz, DMSO-d_6, ppm) δ: 170.6, 148.1, 144.2, 136.4, 132.6, 131.9, 130.8, 130.3, 130.1, 129.9, 128.0, 125.3, 53.2, 40.2, 30.0, 21.5; HRMS (ESI) $[M+Na]^+$ calcd for $C_{18}H_{17}ClN_2O_4S_2$: 447.02105, found: 447.02061.

Data for methyl (*Z*)-6-chloro-4-(2-((4-fluorophenyl)sulfonyl)hydrazineylidene)thiochromane-2-carboxylate (**4i**). White solid; mp 177–179 °C; yield 60.0%; ^{1}H NMR (400 MHz, DMSO-d_6, ppm) δ: 10.99 (s, 1H, NH), 8.02-7.99 (m, 2H, Ph-H), 7.70 (d, J = 2.4 Hz, 1H, Ph-H), 7.50 (t, J = 8.8 Hz, 2H, Ph-H), 7.35-7.28 (m, 2H, Ph-H), 4.42 (q, J1 = 4.8 Hz, J2 = 6.4 Hz, 1H, SCH), 3.63 (s, 3H, CH_3), 3.19-3.04 (m, 2H, CH_2); ^{13}C NMR (100 MHz, DMSO-d_6, ppm) δ: 170.6, 166.4, 163.9, 148.7, 135.6 (d, J = 3.0 Hz), 132.5, 132.0, 131.1, 131.1, 130.9, 130.3, 130.0, 125.3, 117.0, 116.8, 53.2, 40.3, 30.0; HRMS (ESI) $[M+Na]^+$ calcd for $C_{17}H_{15}ClFN_2O_4S_2$: 450.99598, found: 450.99577.

Data for ethyl (*Z*)-6-chloro-4-(2-(phenylsulfonyl)hydrazineylidene)thiochromane-2-carboxylate (**4j**). White solid; mp 190–192 °C; yield 72.0%; ^{1}H NMR (400 MHz, DMSO-d_6, ppm) δ: 10.96 (s, 1H, NH), 7.94 (d, J = 7.2 Hz, 2H, Ph-H), 7.72-7.62 (m, 4H, Ph-H), 7.34-7.27 (m, 2H, Ph-H), 4.38 (q, J = 4.8 Hz, 1H, SCH), 4.09-4.03 (m, 2H, CH_2), 3.18-3.01 (m, 2H, CH_2), 1.10 (t, J = 7.2 Hz, 3H, CH_3); ^{13}C NMR (100 MHz, DMSO-d_6, ppm) δ: 170.1, 148.4, 139.3, 133.8, 132.7, 132.0, 130.8, 130.3, 129.9, 129.6, 128.0, 125.3, 61.9, 40.2, 30.0, 14.3; HRMS (ESI) $[M+Na]^+$ calcd for $C_{18}H_{17}ClN_2O_4S_2$: 447.02105, found: 447.02095.

Data for ethyl (*Z*)-6-chloro-4-(2-tosylhydrazineylidene)thiochromane-2-carboxylate (**4k**). White solid; mp 177–178 °C; yield 74.5%; ^{1}H NMR (400 MHz, DMSO-d_6, ppm) δ: 10.87 (s, 1H, NH), 7.81 (d, J = 8.0 Hz, 2H, Ph-H), 7.69 (d, J = 2.0 Hz, 1H, Ph-H), 7.81 (d, J = 8.0 Hz, 2H, Ph-H), 7.44 (d, J = 8.0 Hz, 2H, Ph-H), 7.34-7.27 (m, 2H, Ph-H), 4.38 (q, J = 4.4 Hz, 1H, SCH), 4.09-4.04 (m, 2H, CH_2), 3.16-3.00 (m, 2H, CH_2), 2.39 (s, 3H, CH_3), 1.10 (t, J = 7.2 Hz, 3H, CH_3); ^{13}C NMR (100 MHz, DMSO-d_6, ppm) δ: 170.1, 148.1, 144.2, 136.4, 132.7, 131.9, 130.8, 130.3, 130.1, 129.9, 128.0, 125.3, 61.9, 40.2, 30.0, 21.5, 14.3; HRMS (ESI) $[M+Na]^+$ calcd for $C_{19}H_{19}ClN_2O_4S_2$: 461.03670, found: 461.03657.

Data for ethyl (*Z*)-6-chloro-4-(2-((4-fluorophenyl)sulfonyl)hydrazineylidene)thiochromane-2-carboxylate (**4l**). White solid; mp 150–152 °C; yield 77.0%; ^{1}H NMR (400 MHz, DMSO-d_6, ppm) δ: 11.02 (s, 1H, NH), 8.05-8.00 (m, 2H, Ph-H), 7.05 (d, J = 2.4 Hz, 1H, Ph-H), 7.53-7.47 (m, 2H, Ph-H), 7.34-7.28 (m, 2H, Ph-H), 4.39 (t, J = 2.0 Hz, 1H, SCH), 4.08 (q, J1 = 1.2 Hz, J2 = 7.2 Hz, 2H, $\underline{CH_2}CH_3$), 3.21-3.04 (m, 2H, CH_2), 1.11 (t, J = 7.2 Hz, 3H, $CH_2\underline{CH_3}$); ^{13}C NMR (100 MHz, DMSO-d_6, ppm) δ: 170.1, 166.4, 163.9, 148.8, 135.6, 132.6, 132.0, 131.1 (d, J = 10.0 Hz), 130.8, 130.3, 129.9, 125.3, 117.0, 116.7, 116.5, 61.9, 40.5, 30.1, 14.3; HRMS (ESI) $[M+Na]^+$ calcd for $C_{18}H_{17}ClFN_2O_4S_2$: 465.01163, found: 465.01122.

Data for methyl (*Z*)-6-methyl-4-(2-(phenylsulfonyl)hydrazineylidene)thiochromane-2-carboxylate (**4m**). White solid; mp 190–192 °C; yield 74.4%; ^{1}H NMR (400 MHz, DMSO-d_6, ppm) δ: 10.71 (s, 1H, NH), 7.96 (s, 1H, Ph-H), 7.95 (d, J = 1.6 Hz, 1H, Ph-H), 7.71-7.62 (m, 3H, Ph-H), 7.57 (s, 1H, Ph-H), 7.13-7.08 (m, 2H, Ph-H), 4.32 (t, J = 5.2 Hz, 1H, SCH), 3.61 (s, 3H, CH_3), 3.08 (q, J1 = 5.2 Hz, J2 = 6.8 Hz, 2H, CH_2), 2.25 (s, 3H, CH_3); ^{13}C NMR (100 MHz, DMSO-d_6, ppm) δ: 170.8, 150.0, 139.3, 135.6, 133.6, 131.2, 130.9, 129.6, 129.5, 128.3, 128.1, 126.5, 52.1, 40.4, 30.6, 21.2; HRMS (ESI) $[M+Na]^+$ calcd for $C_{18}H_{18}N_2O_4S_2$: 413.06002, found: 413.06136.

Data for methyl (*Z*)-6-methyl-4-(2-tosylhydrazineylidene)thiochromane-2-carboxylate (**4n**). White solid; mp 182–184 °C; yield 78.5%; ^{1}H NMR (400 MHz, DMSO-d_6, ppm) δ: 10.62 (s,

1H, NH), 7.83 (d, J = 8.0 Hz, 2H, Ph-H), 7.59 (s, 1H, Ph-H), 7.43 (d, J = 8.0 Hz, 2H, Ph-H), 7.13-7.08 (m, 2H, Ph-H), 4.31 (t, J = 5.6 Hz, 1H, SCH), 3.61 (s, 3H, CH_3), 3.06 (t, J = 2.8 Hz, 2H, CH_2), 2.38 (s, 3H, CH_3), 2.26 (s, 3H, CH_3); ^{13}C NMR (100 MHz, DMSO-d_6, ppm) δ: 170.8, 149.7, 144.0, 136.5, 135.6, 131.2, 130.9, 129.9, 129.6, 128.3, 128.2, 126.5, 53.1, 40.6, 30.7, 21.5, 21.2; HRMS (ESI) $[M+Na]^+$ calcd for $C_{19}H_{20}N_2O_4S_2$: 427.07567, found: 427.07663.

Data for methyl (*Z*)-4-(2-((4-fluorophenyl)sulfonyl)hydrazineylidene)-6-methylthiochromane-2-carboxylate (**4o**). White solid; mp 180–181 °C; yield 80.5%; ^1H NMR (400 MHz, DMSO-d_6, ppm) δ: 10.73 (s, 1H, NH), 8.04-8.00 (m, 2H, Ph-H), 7.58 (s, 1H, Ph-H), 7.52-7.47 (m, 2H, Ph-H), 7.14-7.09 (m, 2H, Ph-H), 4.33 (t, J = 5.2 Hz, 1H, SCH), 3.62 (s, 3H, CH_3), 3.14-3.04 (m, 2H, CH_2), 2.27 (s, 3H, CH_3); ^{13}C NMR (100 MHz, DMSO-d_6, ppm) δ: 170.8, 163.8, 150.4, 135.7 (d, J = 2.0 Hz), 131.3, 131.2, 130.8, 129.7, 128.3, 126.5, 116.9, 116.7, 53.1, 40.5, 30.7, 21.2; HRMS (ESI) $[M+Na]^+$ calcd for $C_{18}H_{18}FN_2O_4S_2$: 431.05060, found: 431.05046.

Data for ethyl (*Z*)-6-methyl-4-(2-(phenylsulfonyl)hydrazineylidene)thiochromane-2-carboxylate (**4p**). White solid; mp 196–198 °C; yield 75.5%; ^1H NMR (400 MHz, DMSO-d_6, ppm) δ: 10.71 (s, 1H, NH), 7.97-7.95 (m, 2H, Ph-H), 7.71-7.62 (m, 3H, Ph-H), 7.57 (s, 1H, Ph-H), 7.13-7.07 (m, 2H, Ph-H), 4.29 (q, J1 = 5.2 Hz, J2 = 6.8 Hz, 1H, SCH), 4.07-4.03 (m, 2H, $\underline{CH_2}CH_3$), 3.14-3.06 (m, 2H, CH_2), 1.10 (t, J = 6.8 Hz, 3H, $CH_2\underline{CH_3}$); ^{13}C NMR (100 MHz, DMSO-d_6, ppm) δ: 170.3, 150.1, 139.3, 135.6, 133.6, 131.2, 130.9, 129.7, 129.5, 128.3, 128.1, 126.5, 61.8, 40.6, 30.7, 21.2, 14.3; HRMS (ESI) $[M+Na]^+$ calcd for $C_{19}H_{20}N_2O_4S_2$: 427.07567, found: 427.07583.

Data for ethyl (*Z*)-6-methyl-4-(2-tosylhydrazineylidene)thiochromane-2-carboxylate (**4q**). White solid; mp 175–176 °C; yield 71.5%; ^1H NMR (400 MHz, DMSO-d_6, ppm) δ: 10.62 (s, 1H, NH), 7.84 (d, J = 8.4 Hz, 2H, Ph-H), 7.58 (s, 1H, Ph-H), 7.43 (d, J = 8.0 Hz, 2H, Ph-H), 7.13-7.07 (m, 2H, Ph-H), 4.28 (q, J1 = 5.2 Hz, J2 = 6.4 Hz, 1H, SCH), 4.08-4.03 (m, 2H, $\underline{CH_2}CH_3$), 3.12-3.00 (m, 2H, CH_2), 2.38 (s, 3H, CH_3), 2.26 (s, 3H, CH_3), 1.10 (t, J = 5.2 Hz, 3H, $CH_2\underline{CH_3}$); ^{13}C NMR (100 MHz, DMSO-d_6, ppm) δ: 170.3, 149.8, 144.0, 136.5, 135.6, 131.1, 131.0, 129.9, 129.6, 128.3, 128.2, 126.5, 61.8, 40.6, 30.7, 21.5, 21.2, 14.3; HRMS (ESI) $[M+Na]^+$ calcd for $C_{20}H_{21}N_2O_4S_2$: 441.09132, found: 441.09179.

Data for ethyl (*Z*)-4-(2-((4-fluorophenyl)sulfonyl)hydrazono)-6-methylthiochromane-2-carboxylate (**4r**). White solid; mp 185–186 °C; yield 78.6%; ^1H NMR (400 MHz, DMSO-d_6, ppm) δ: 10.73 (s, 1H, NH), 8.04-8.01 (m, 2H, Ph-H), 7.58 (s, 1H, Ph-H), 7.49 (t, J = 8.8 Hz, 2H, Ph-H), 7.11 (dd, J1 = 8.0 Hz, J2 = 12.8 Hz, 2H, Ph-H), 4.30 (dd, J1 = 5.2 Hz, J2 = 6.4 Hz, 1H, SCH), 4.06 (q, J1 = 7.2 Hz, J2 = 17.0 Hz, 2H, $\underline{CH_2}CH_3$), 3.15-3.02 (m, 2H, CH_2), 2.26 (s, 3H, CH_3), 1.11 (t, J = 6.8 Hz, 3H, $CH_2\underline{CH_3}$); ^{13}C NMR (100 MHz, DMSO-d_6, ppm) δ: 170.3, 166.3, 163.8, 150.5, 135.6 (d, J = 2.0 Hz), 131.3, 131.2, 130.9, 129.7, 128.3, 126.5, 116.9, 116.6, 61.8, 40.7, 30.7, 21.2, 14.3; HRMS (ESI) $[M+Na]^+$ calcd for $C_{19}H_{19}N_2O_4S_2$: 445.06625, found: 445.06665.

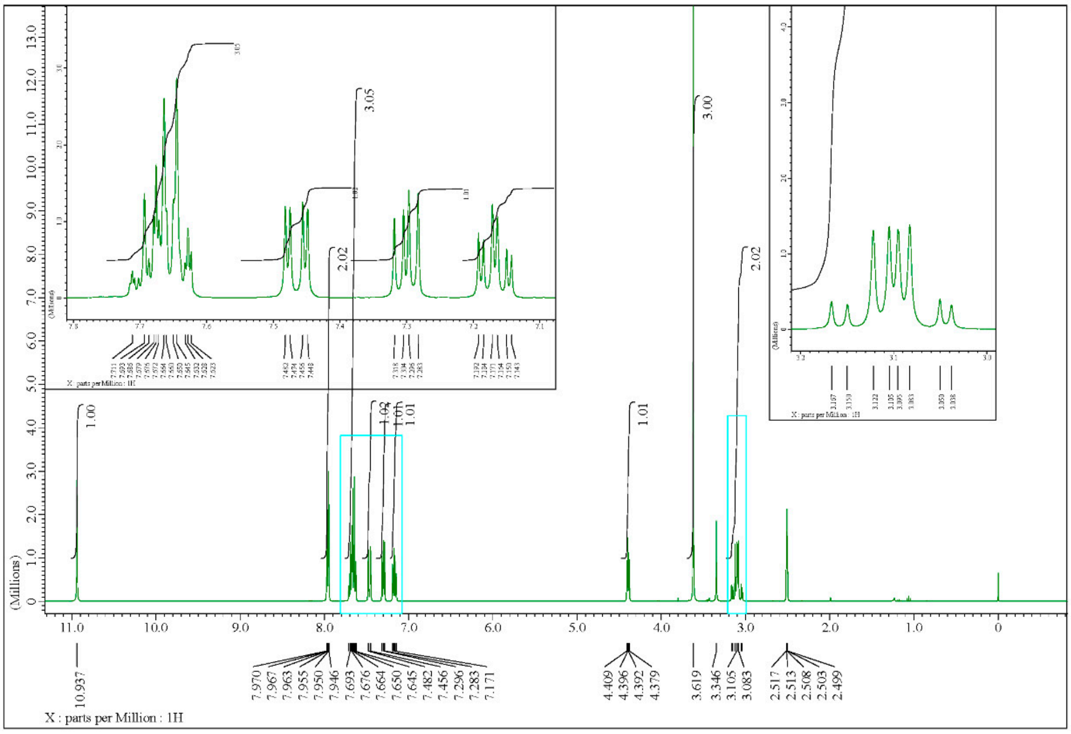

^{1}H NMR of compound **4a**

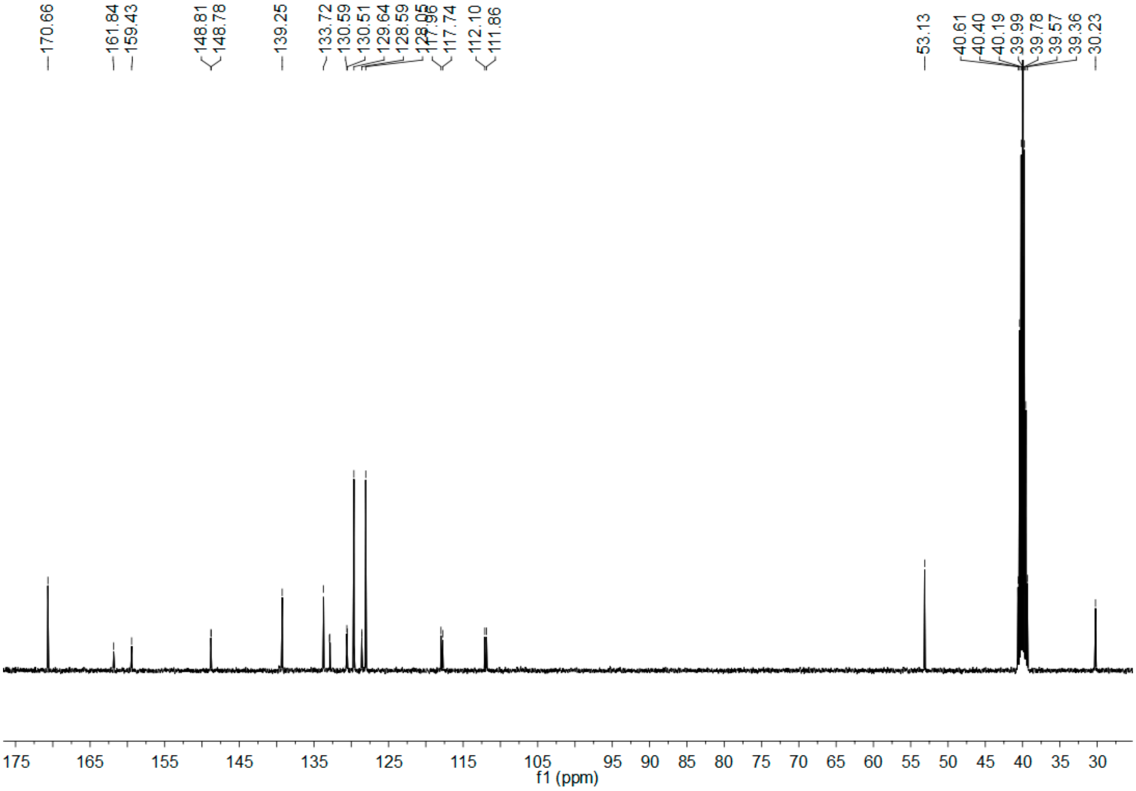

^{13}C NMR of compound **4a**

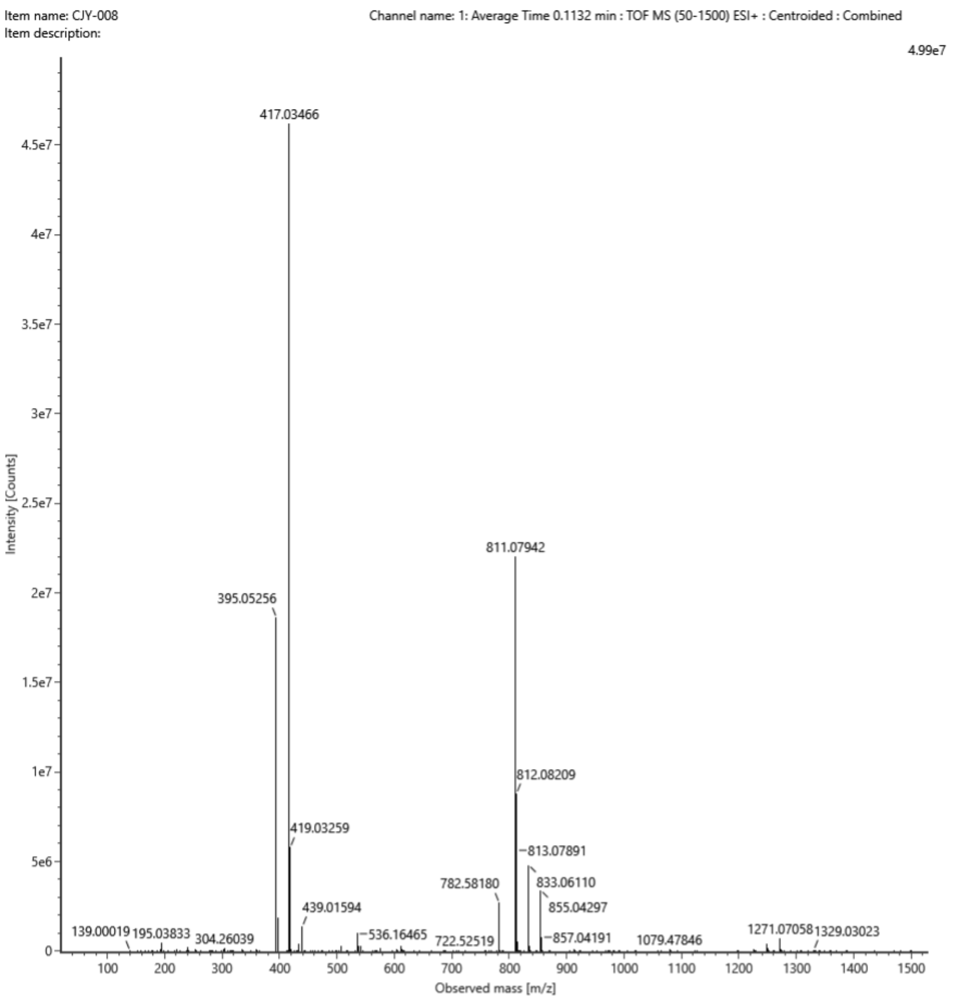

HRMS (ESI) of compound **4a**

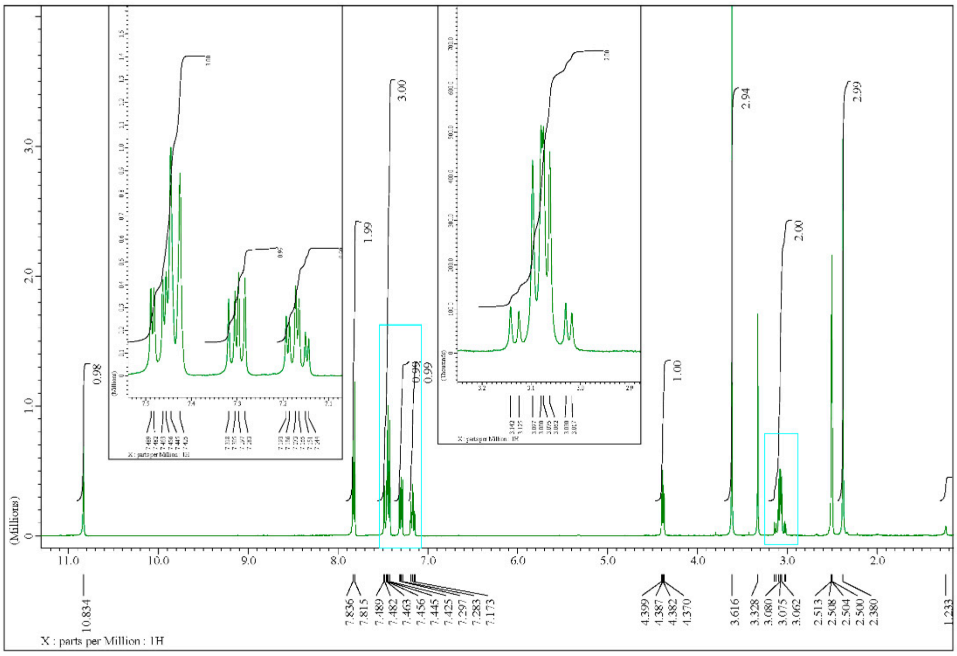

^{1}H NMR of compound **4b**

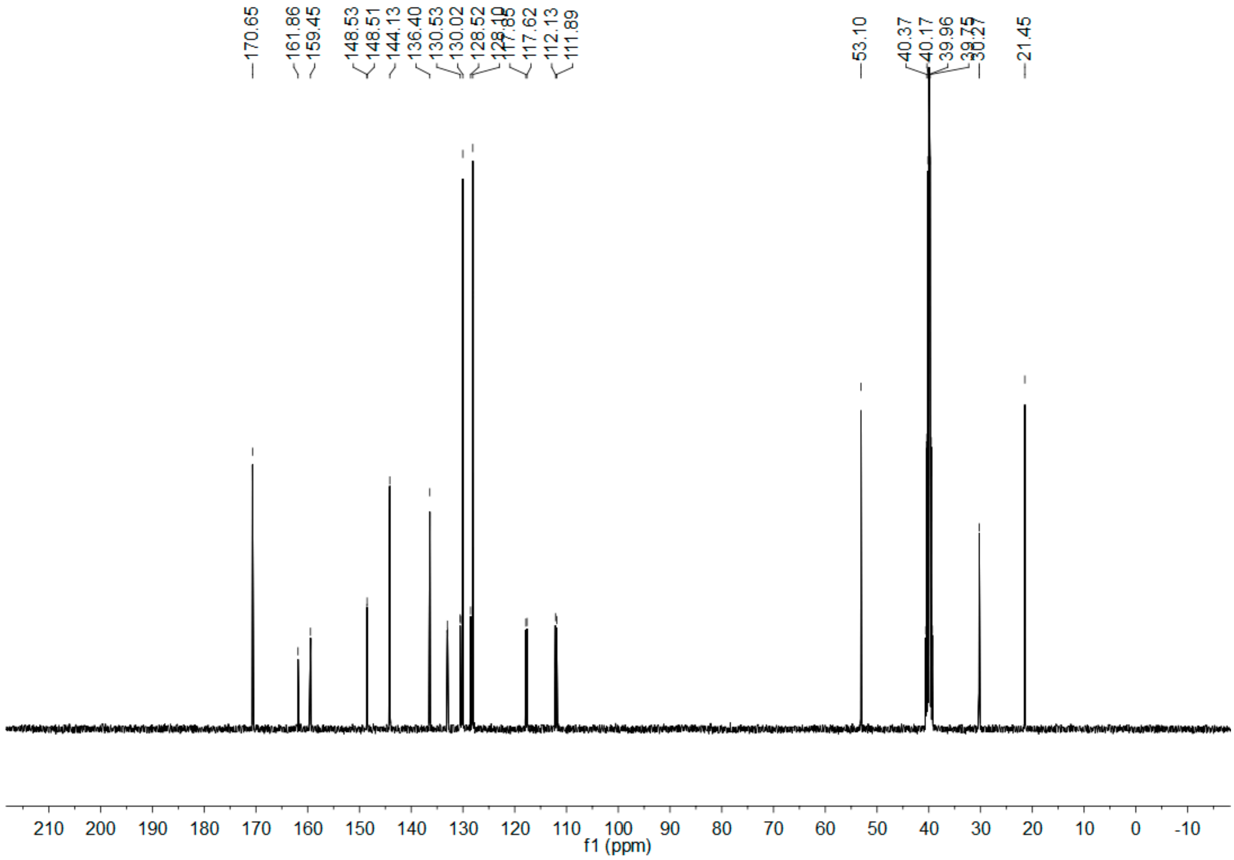

^{13}C NMR of compound **4b**

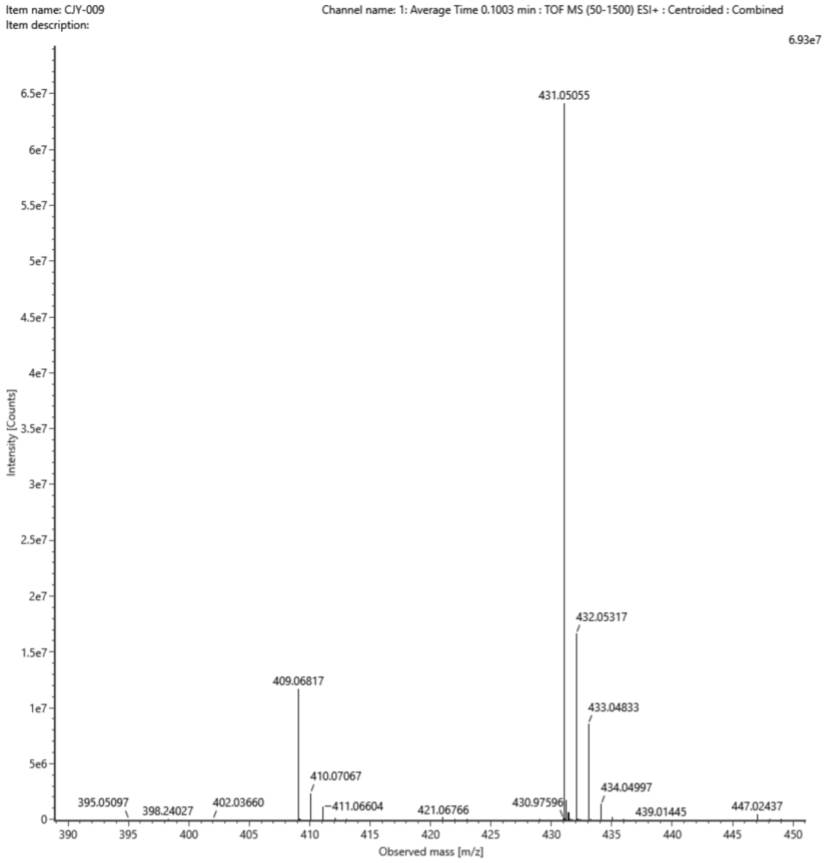

HRMS (ESI) of compound **4b**

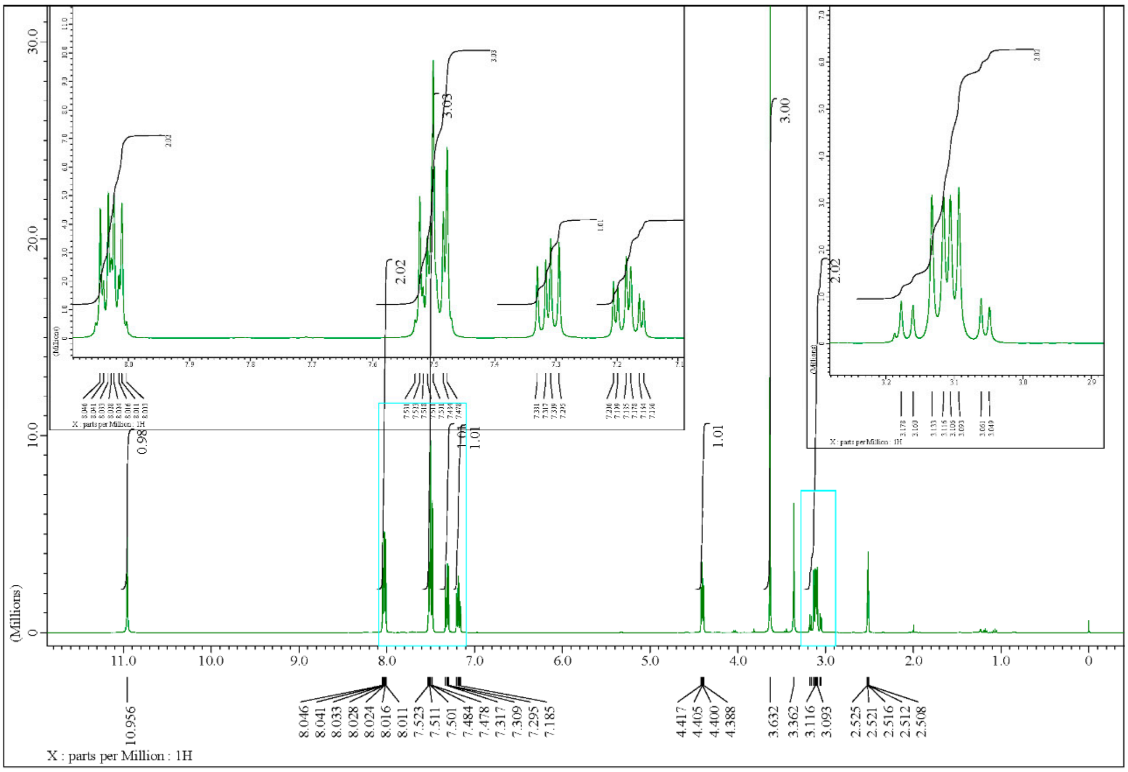

^{1}H NMR of compound **4c**

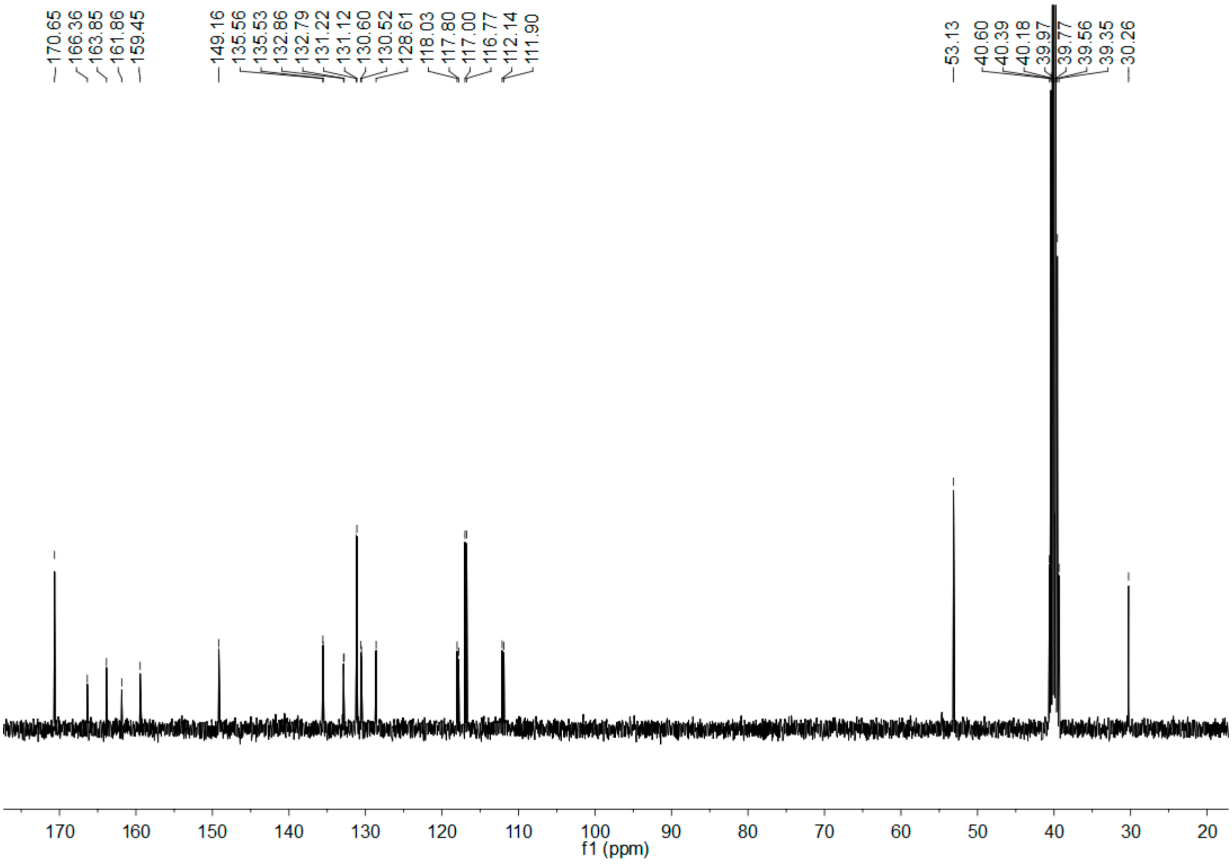

^{13}C NMR of compound **4c**

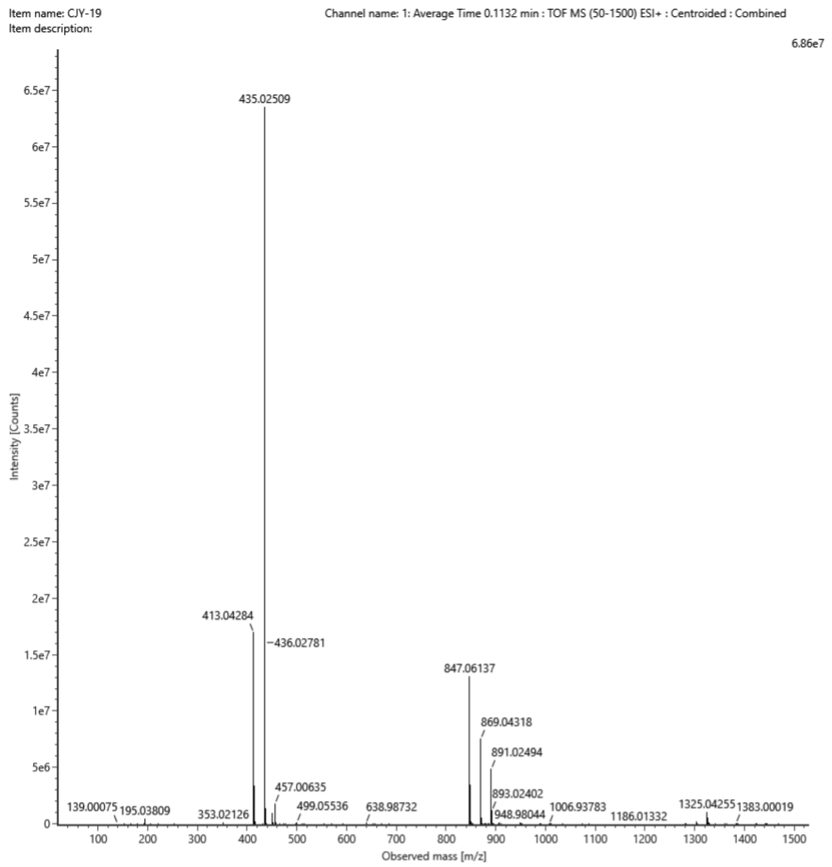

HRMS (ESI) of compound **4c**

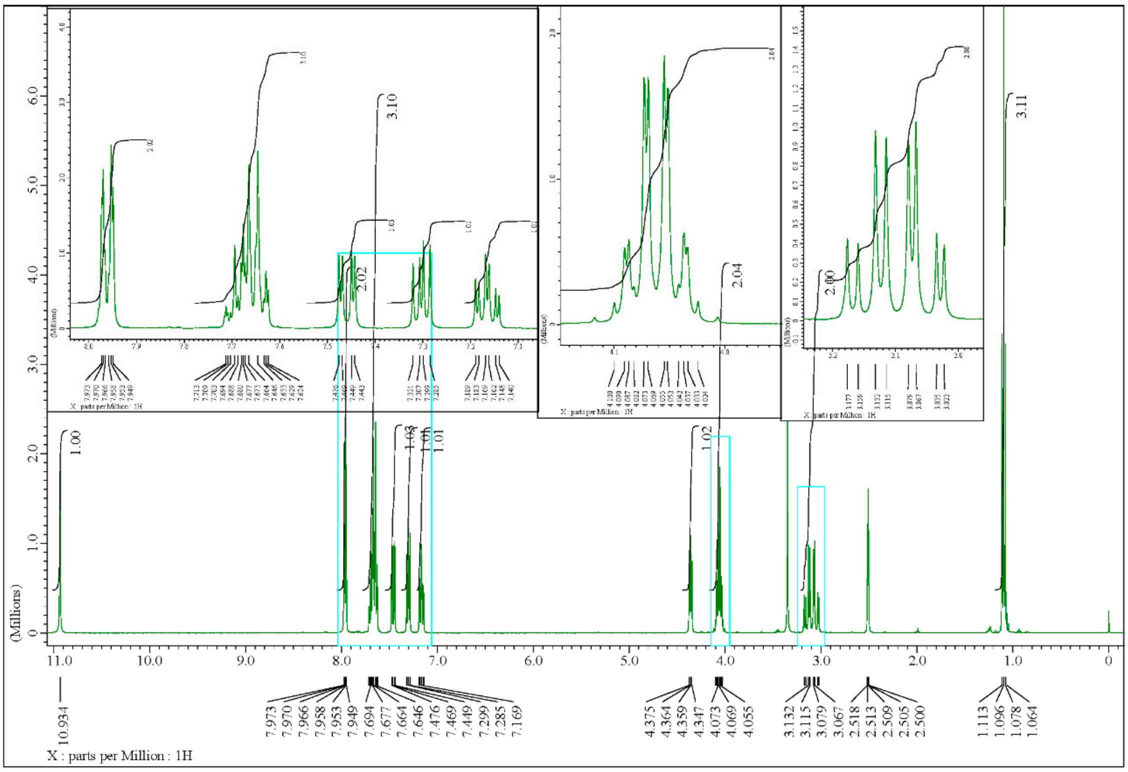

^{1}H NMR of compound **4d**

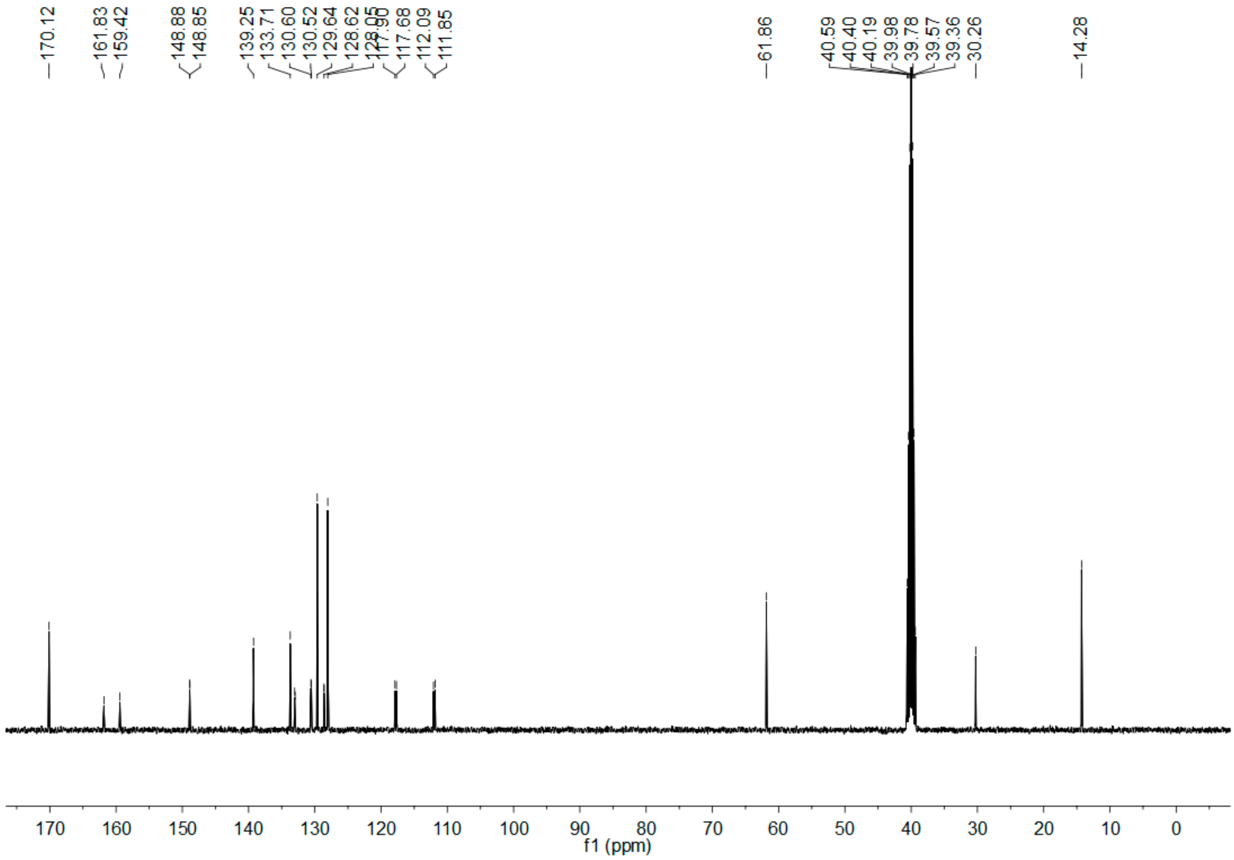

^{13}C NMR of compound **4d**

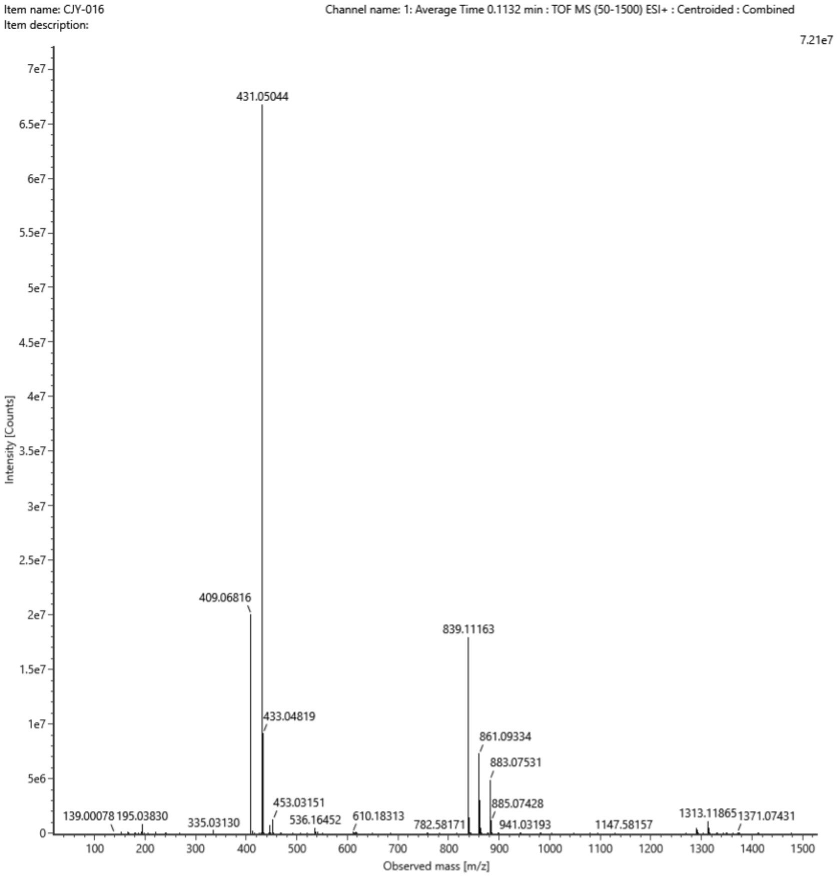

HRMS (ESI) of compound **4d**

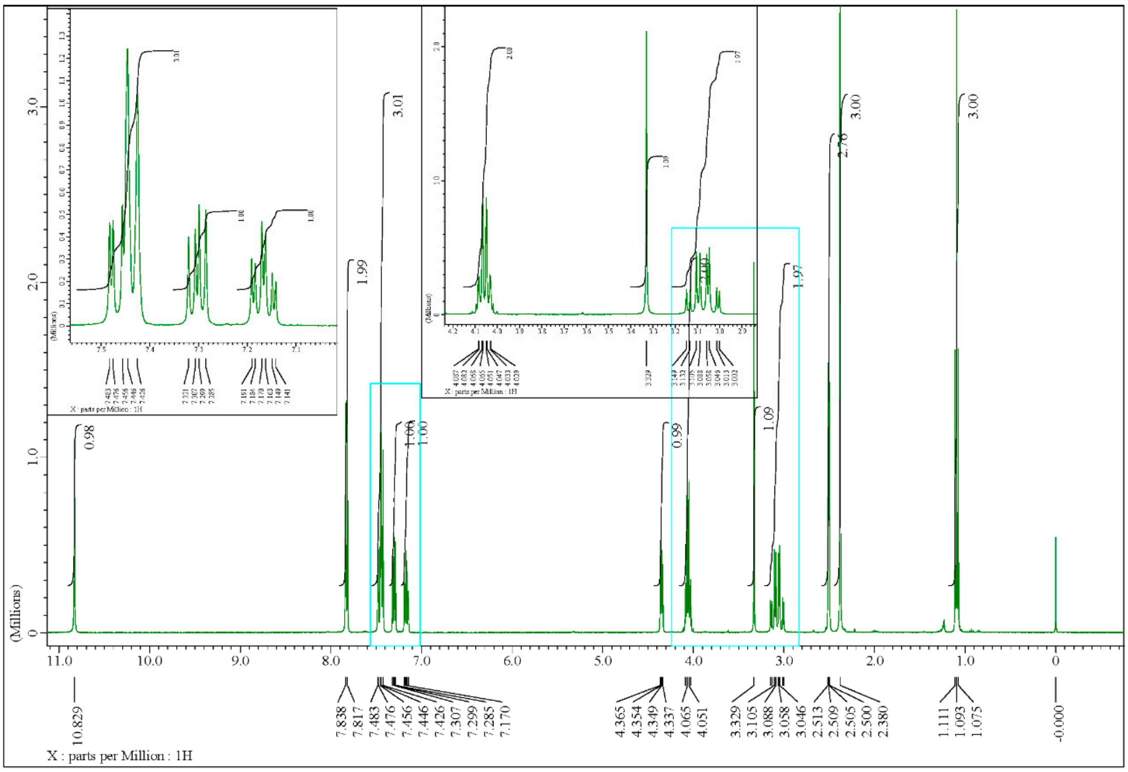

^{1}H NMR of compound **4e**

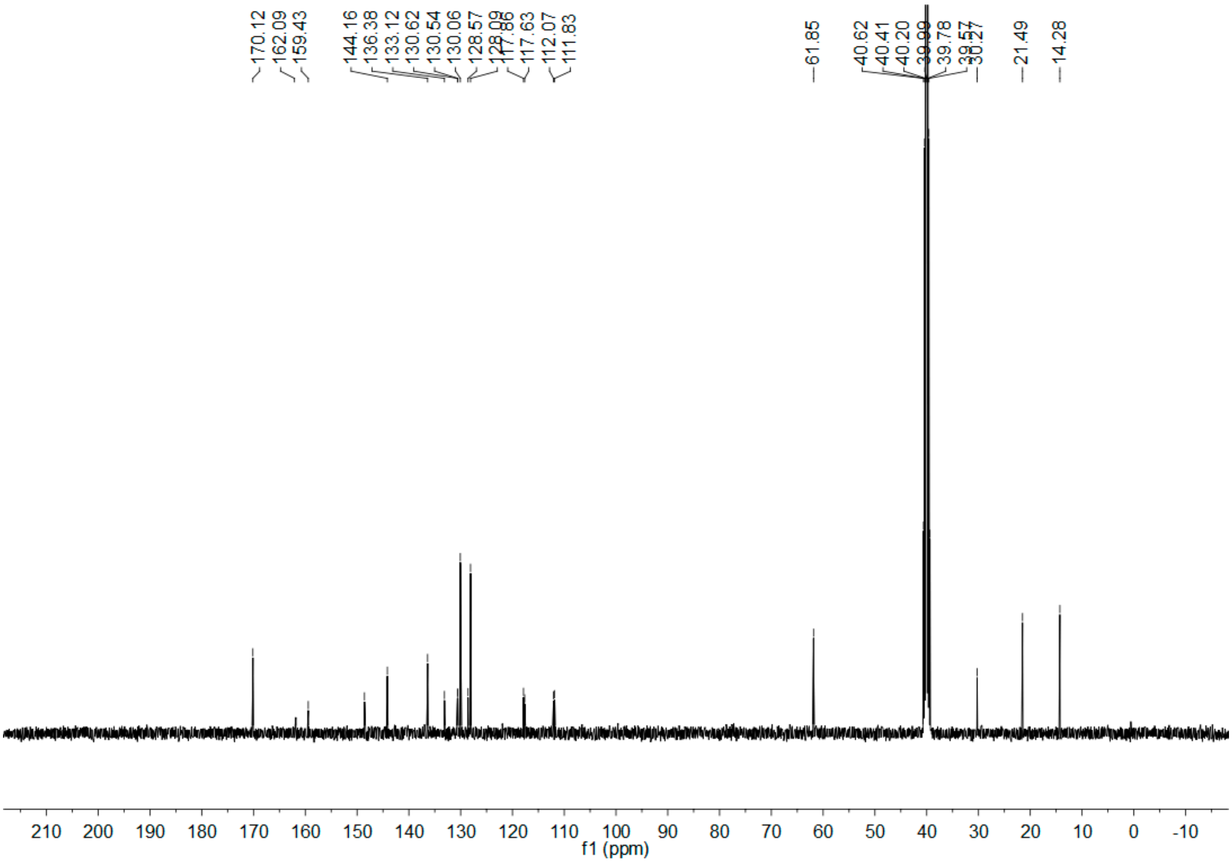

^{13}C NMR of compound **4e**

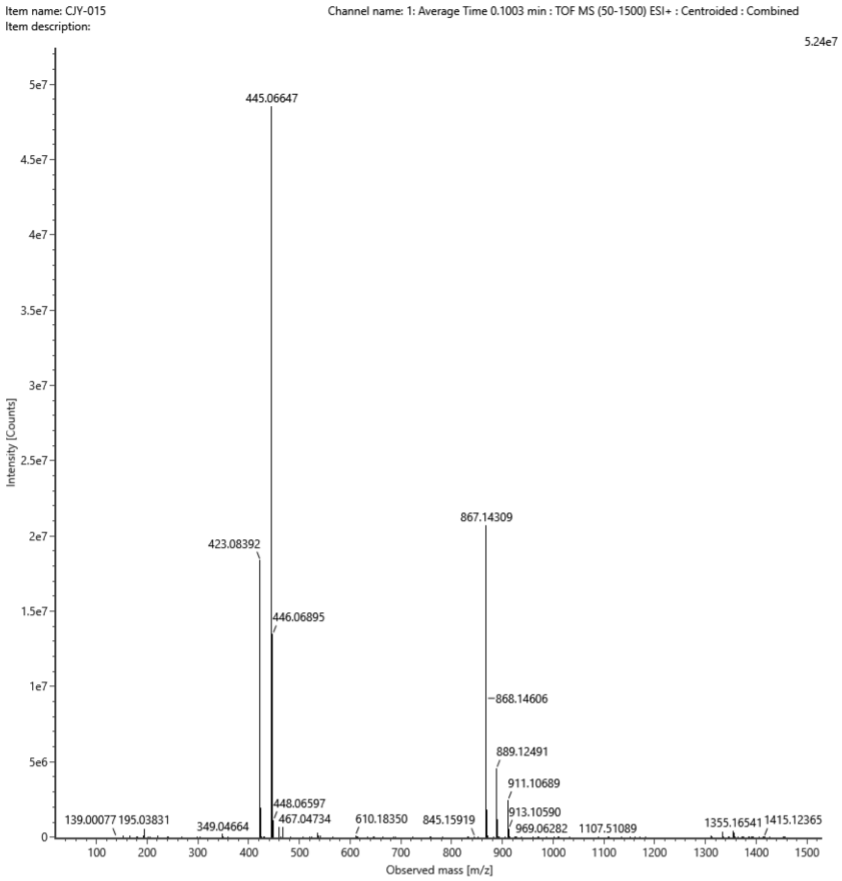

HRMS (ESI) of compound **4e**

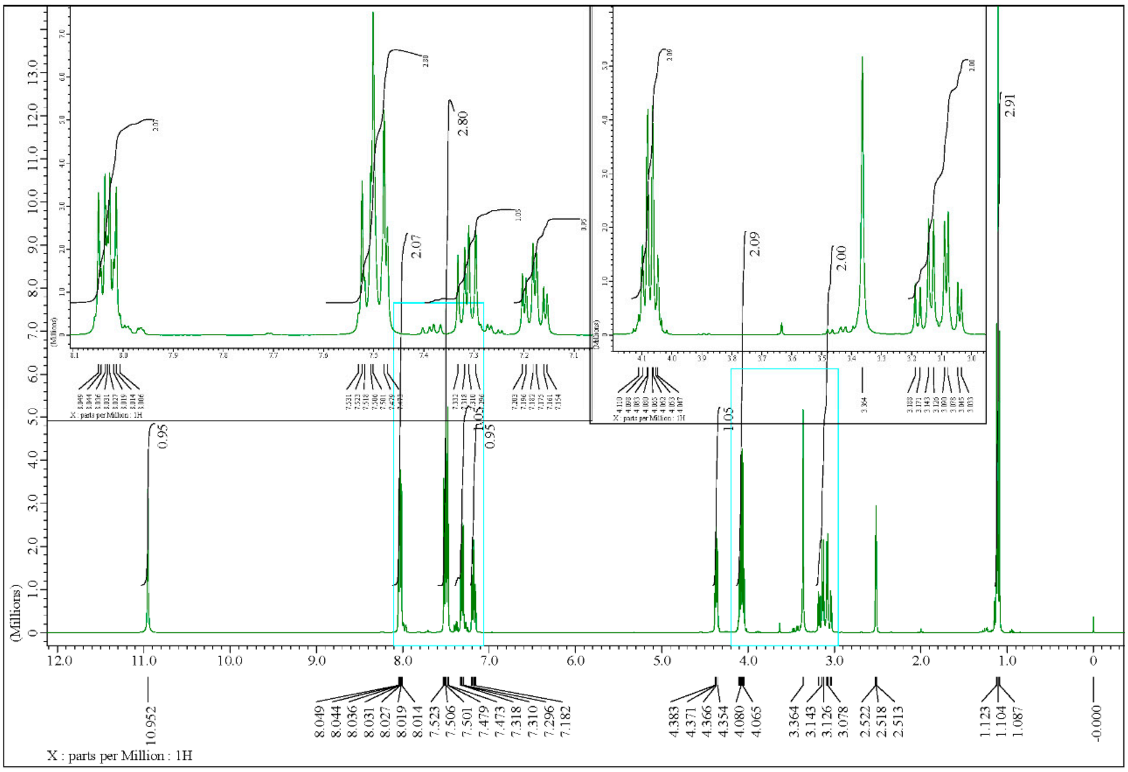

^{1}H NMR of compound **4f**

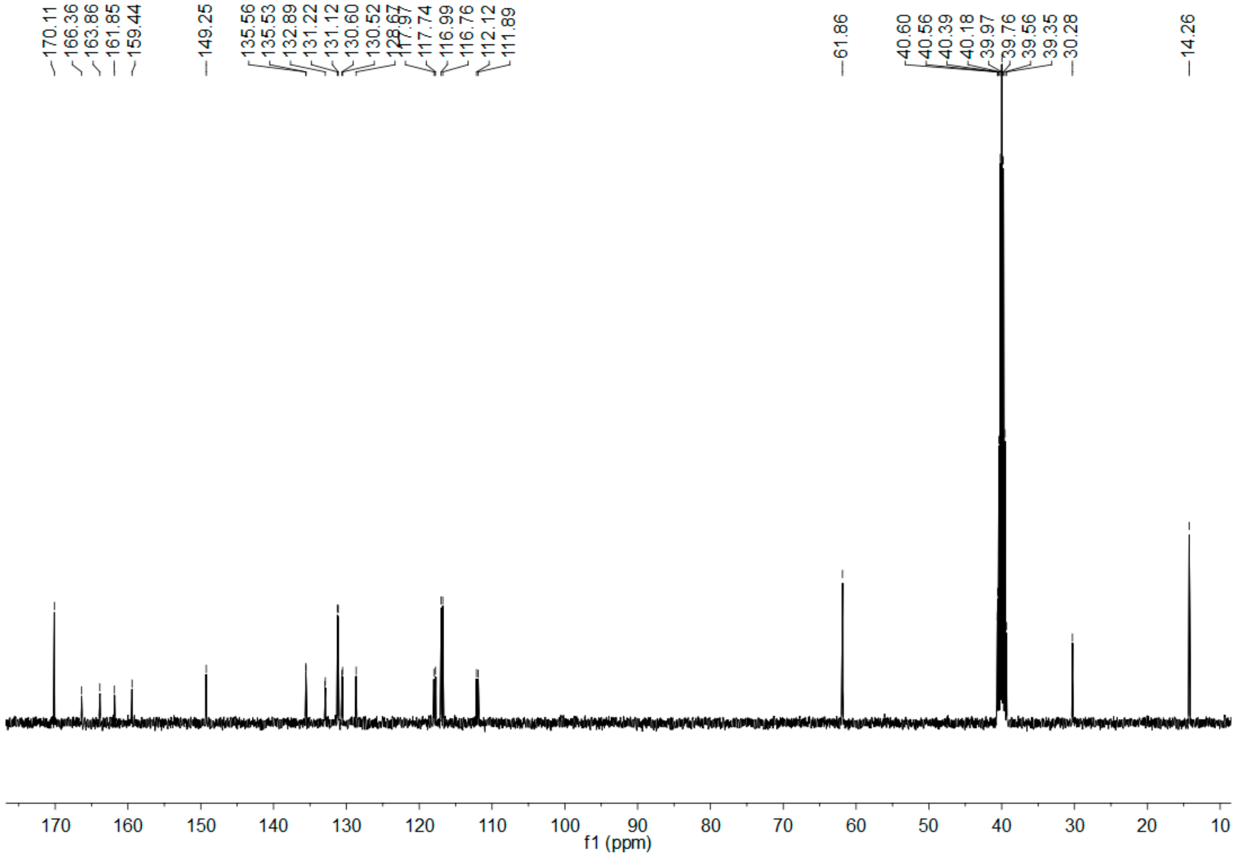

^{13}C NMR of compound **4f**

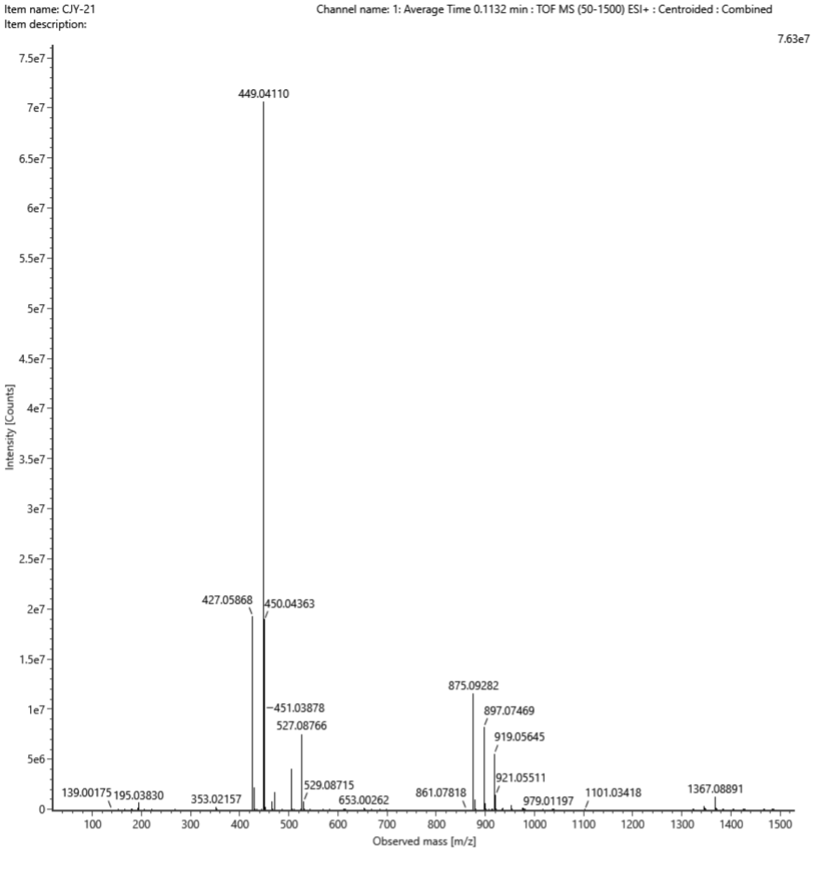

HRMS (ESI) of compound **4f**

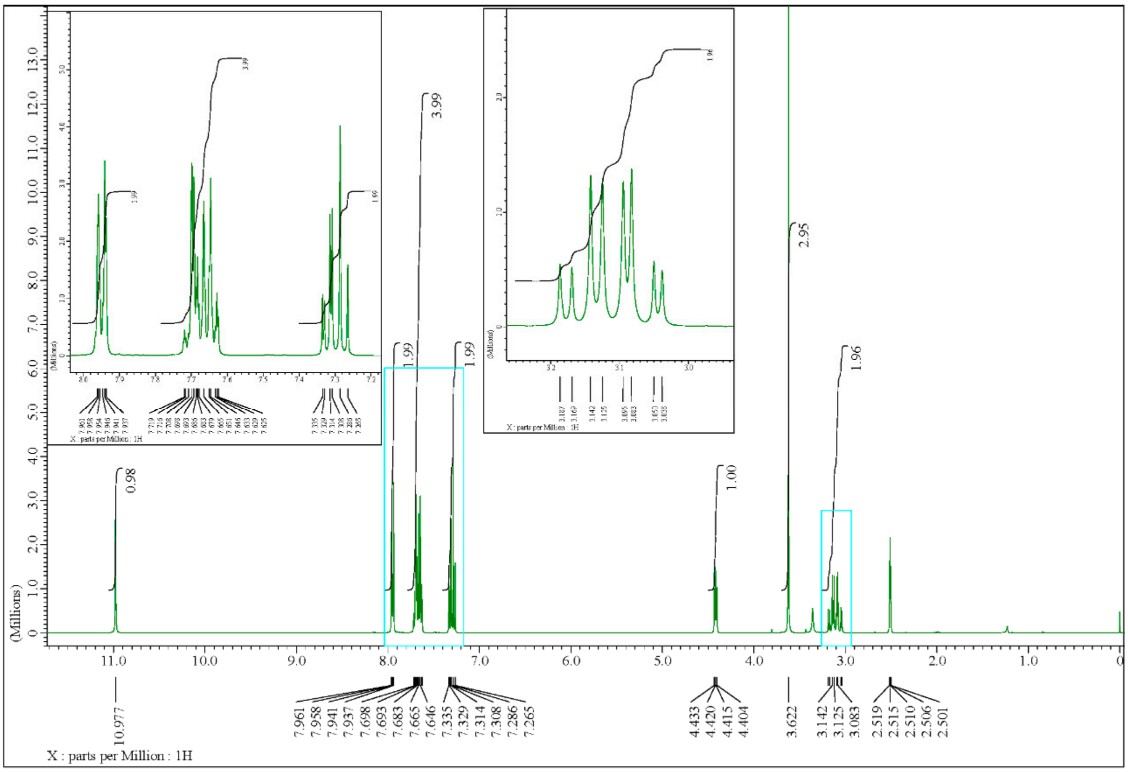

^{1}H NMR of compound **4g**

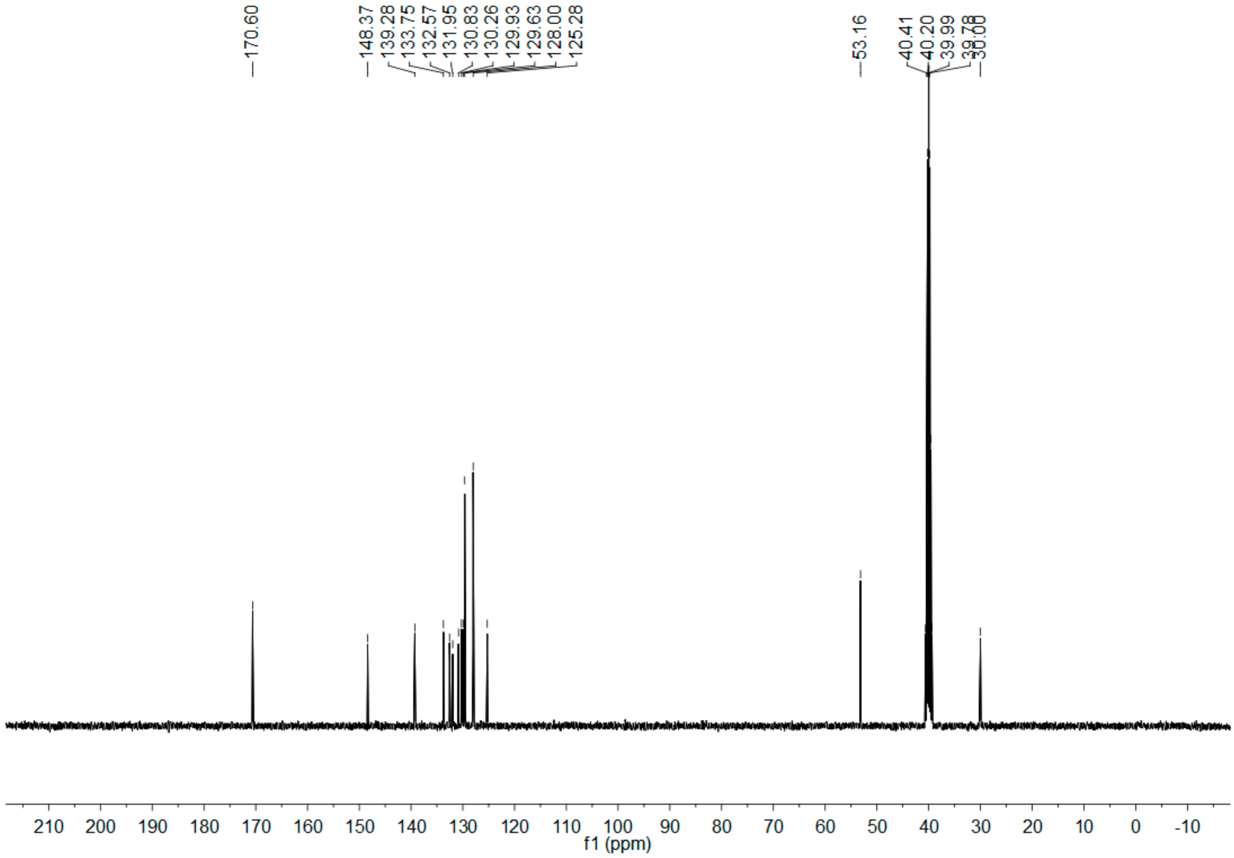

^{13}C NMR of compound **4g**

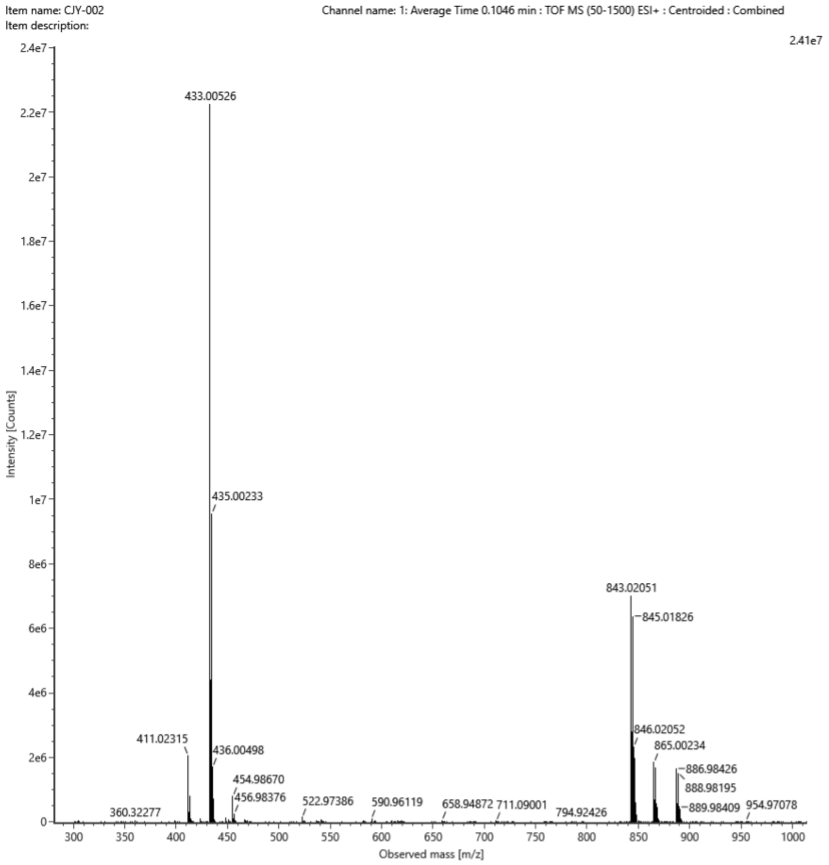

HRMS (ESI) of compound **4g**

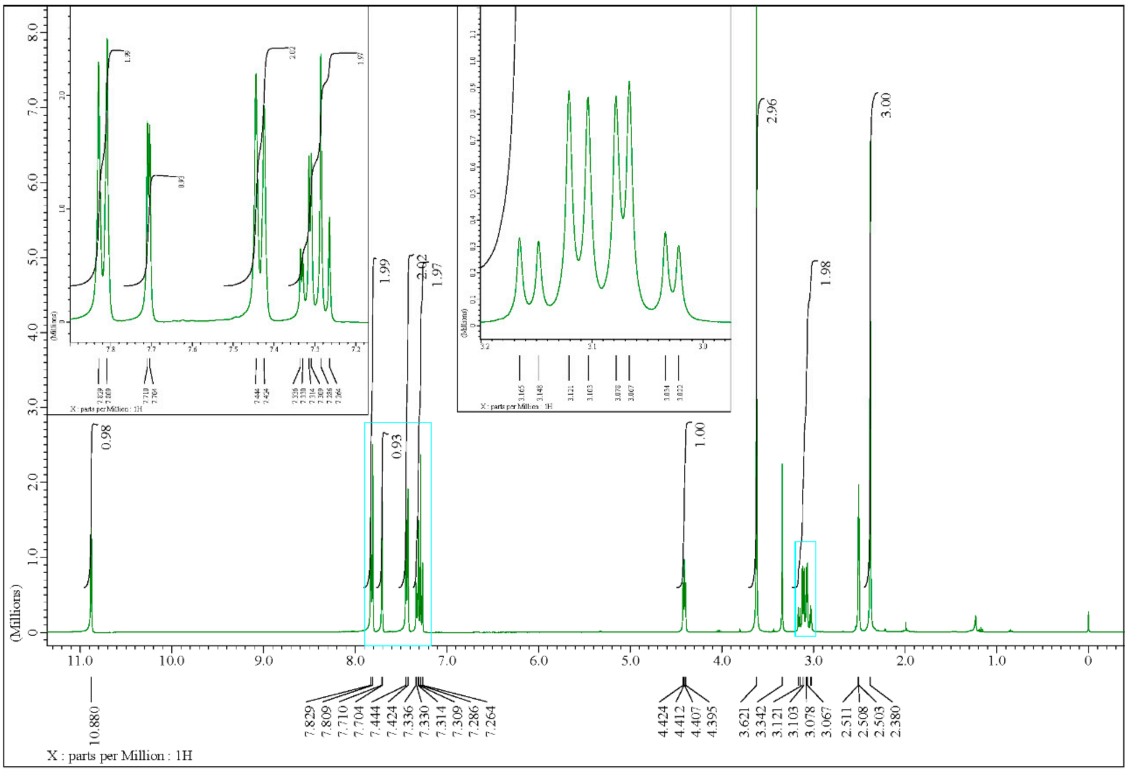

^{1}H NMR of compound **4h**

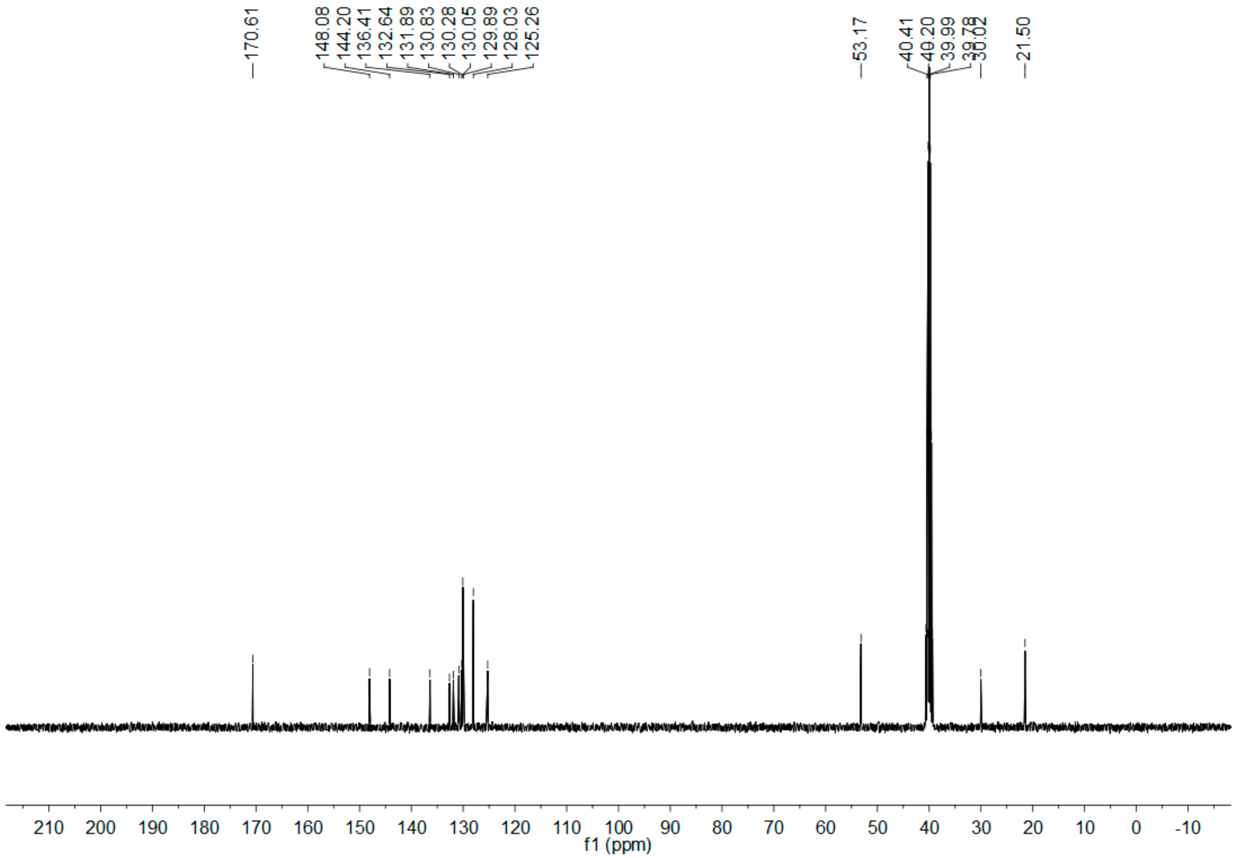

^{13}C NMR of compound **4h**

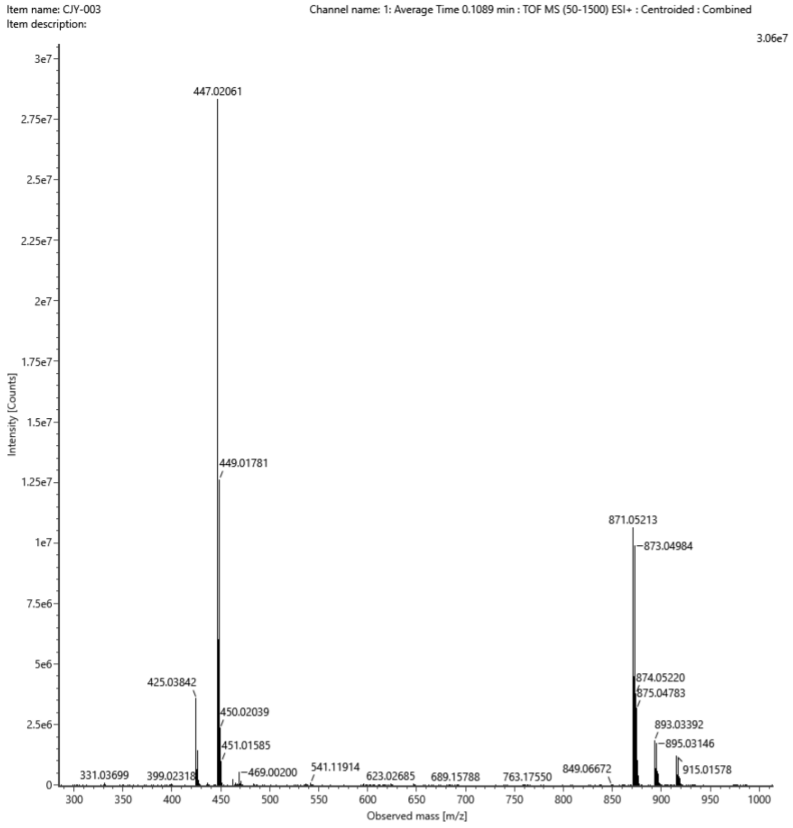

HRMS (ESI) of compound **4h**

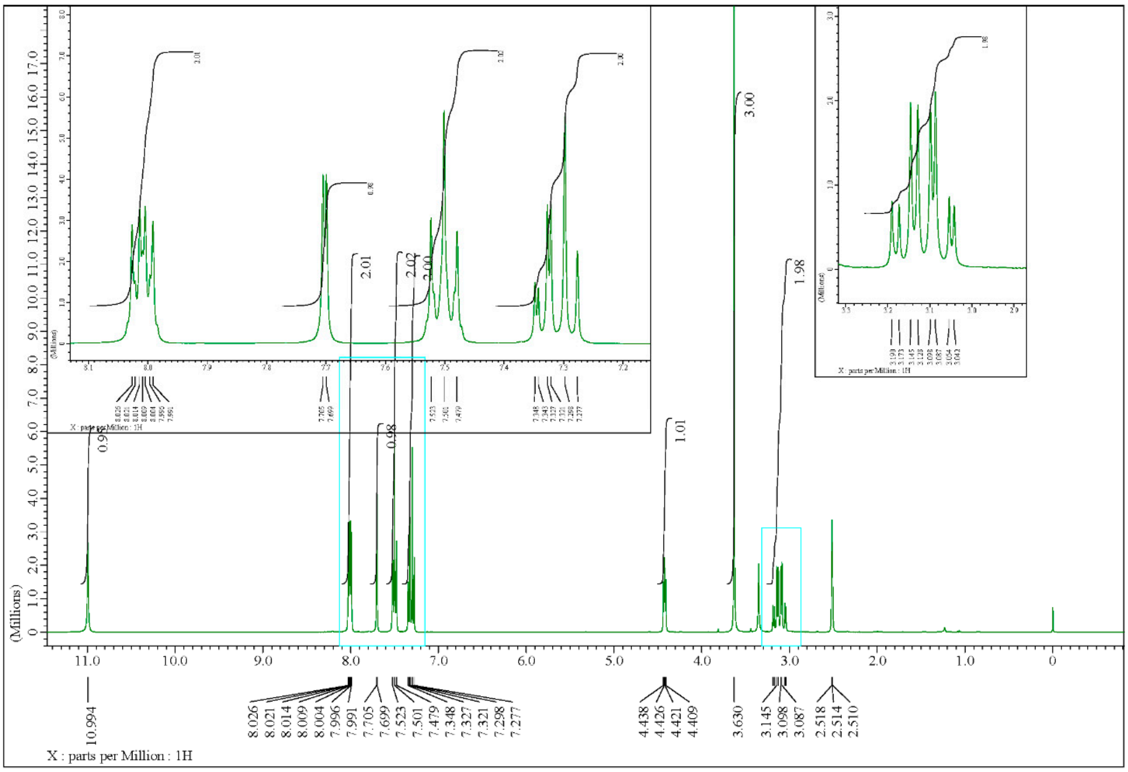

^{1}H NMR of compound **4i**

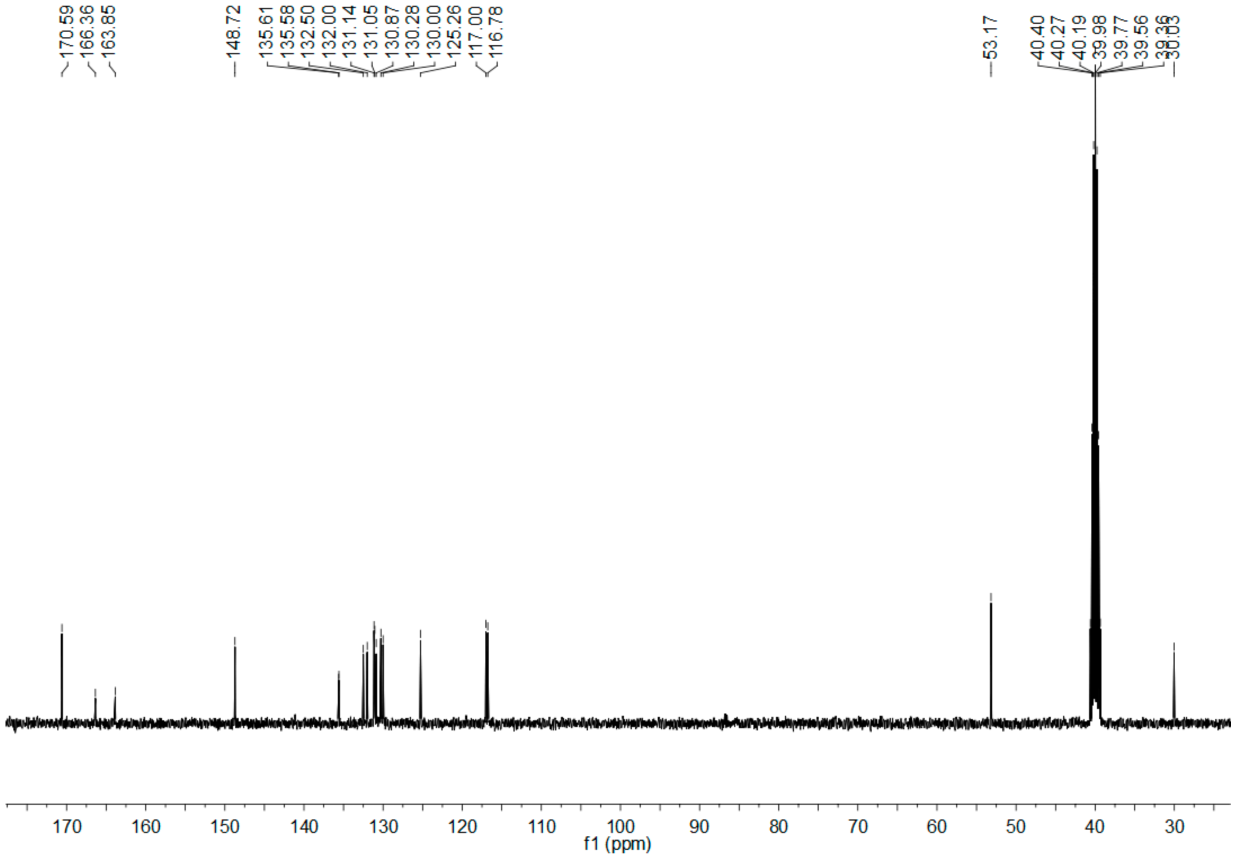

^{13}C NMR of compound **4i**

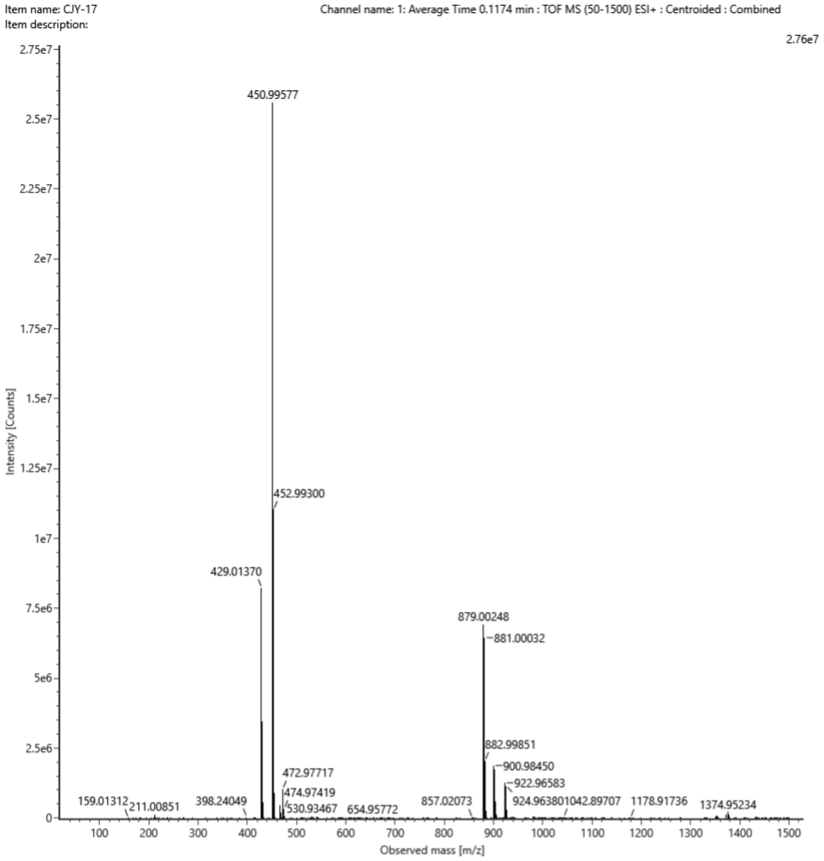

HRMS (ESI) of compound **4i**

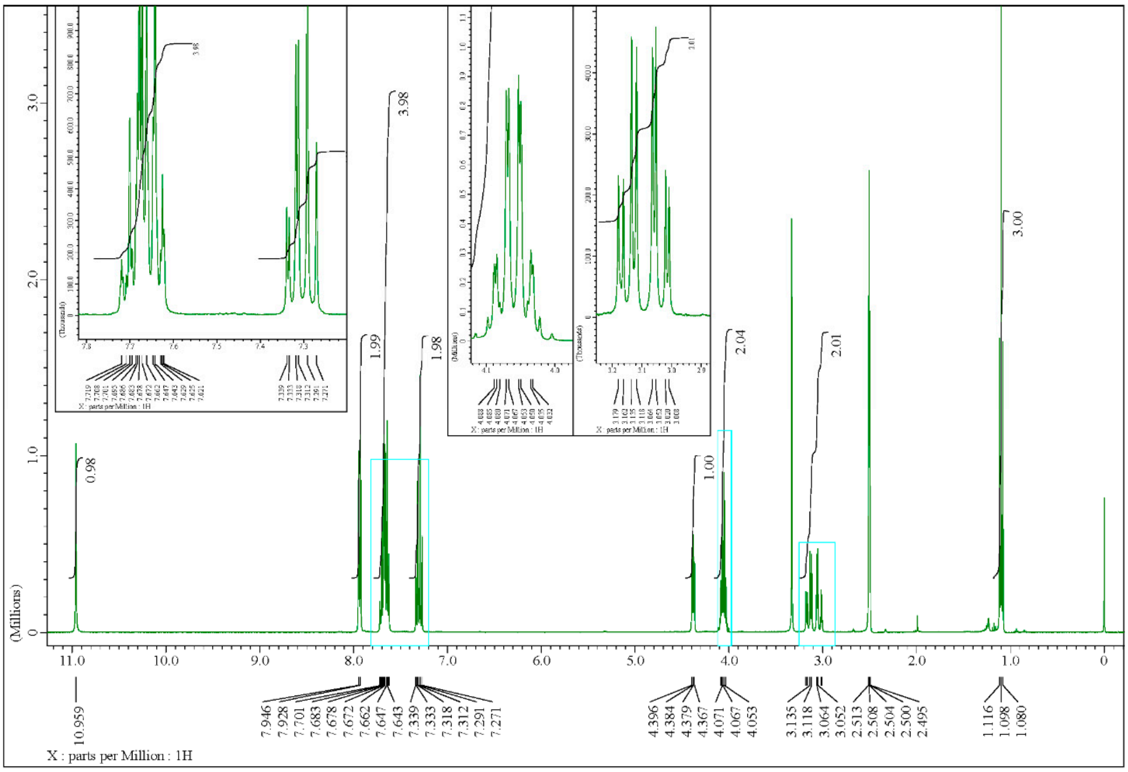

^{1}H NMR of compound **4j**

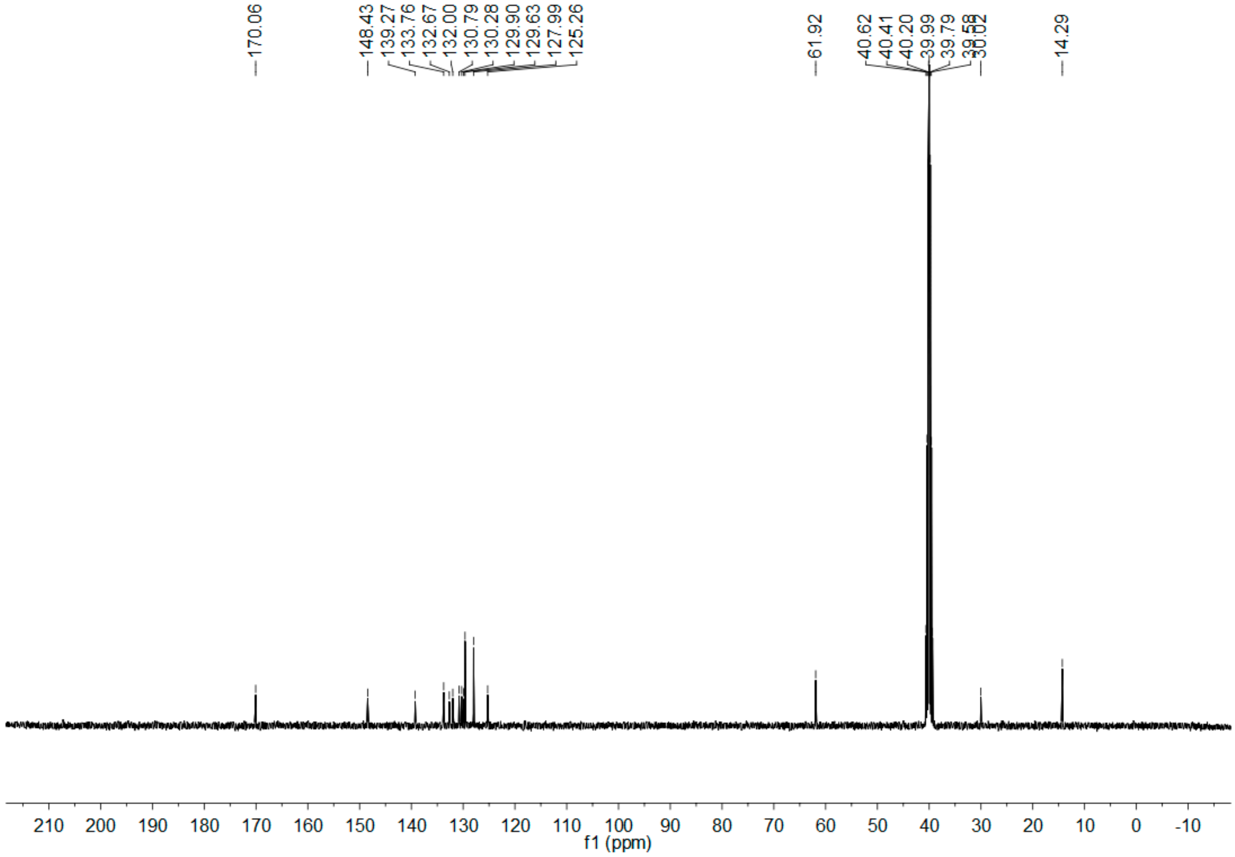

^{13}C NMR of compound **4j**

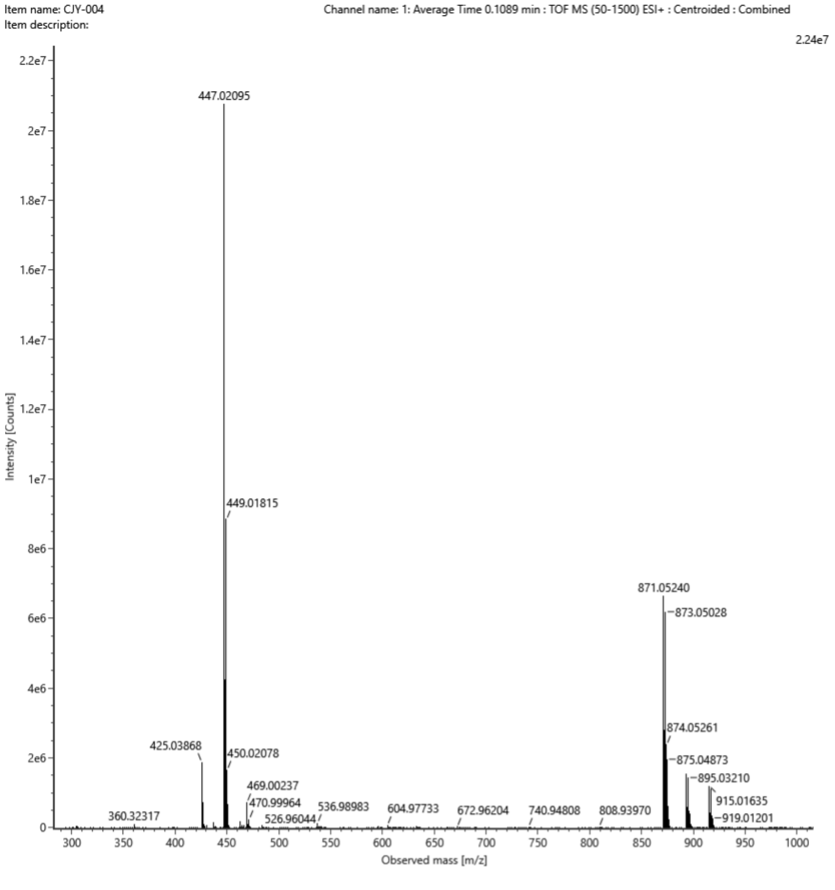

HRMS (ESI) of compound **4j**

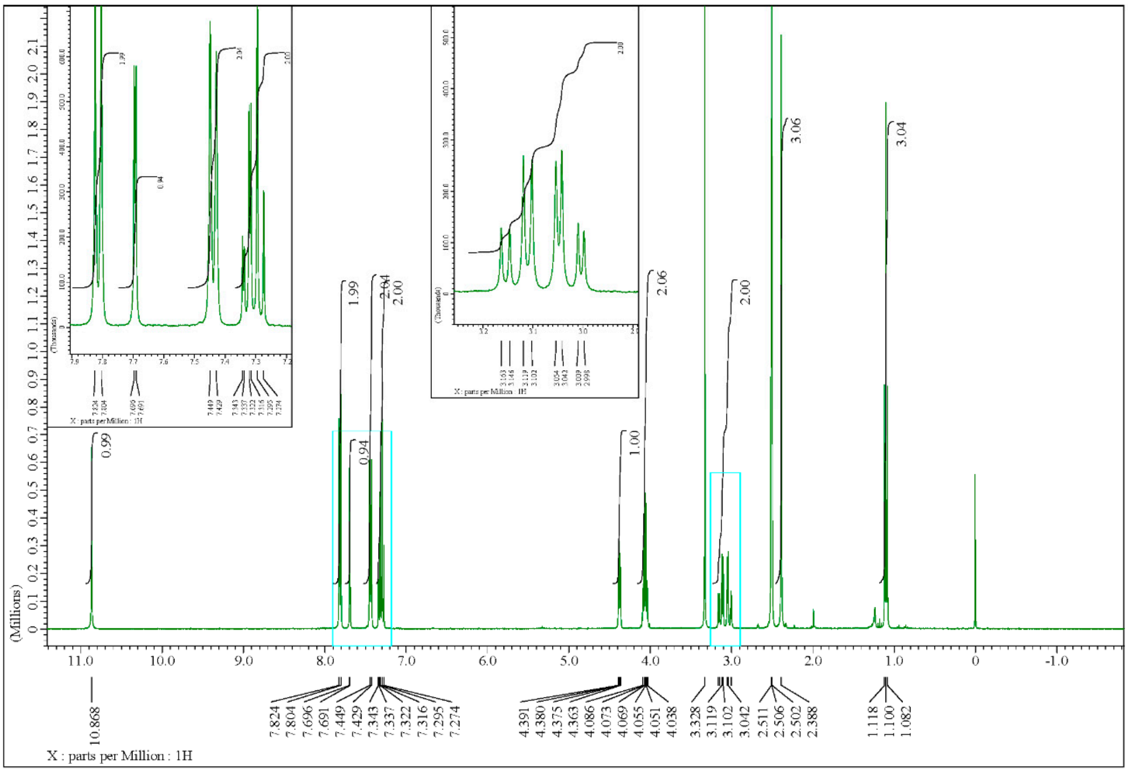

^{1}H NMR of compound **4k**

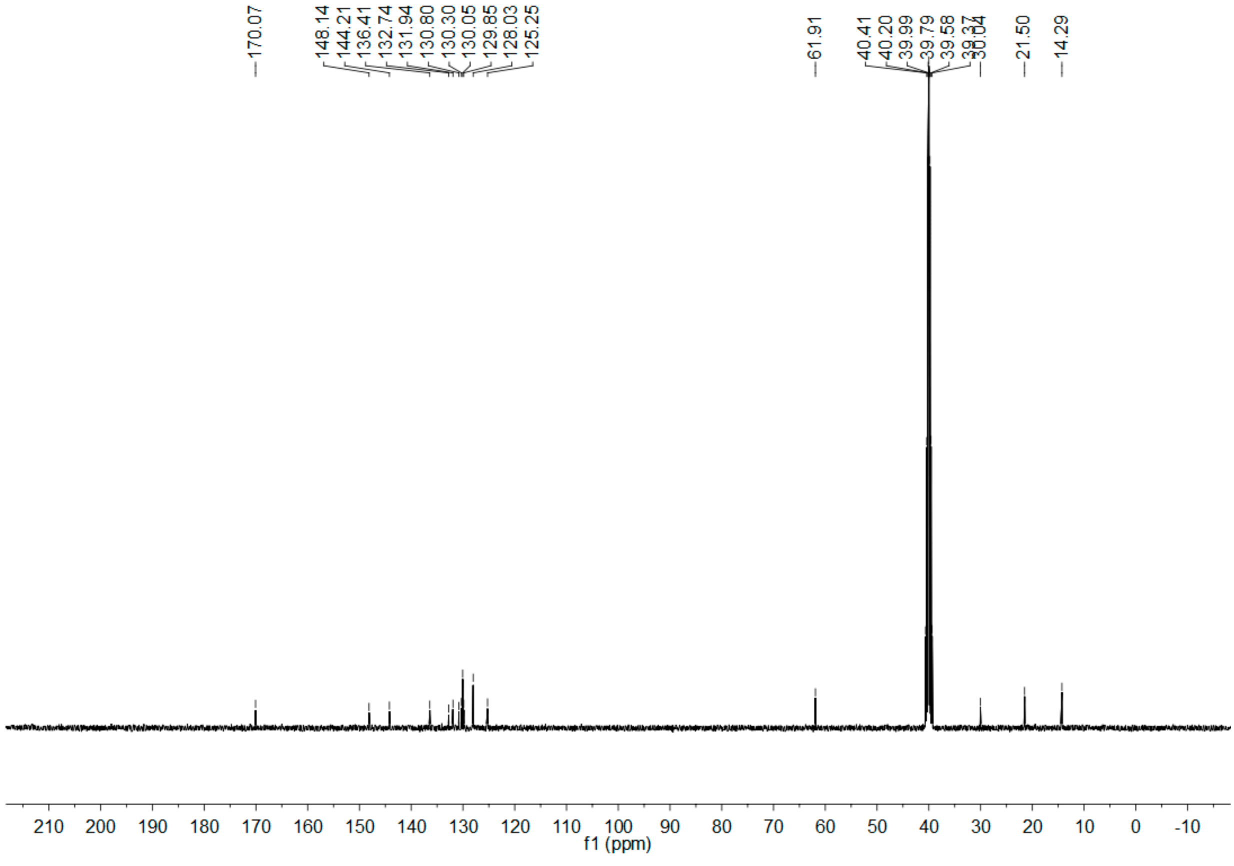

^{13}C NMR of compound **4k**

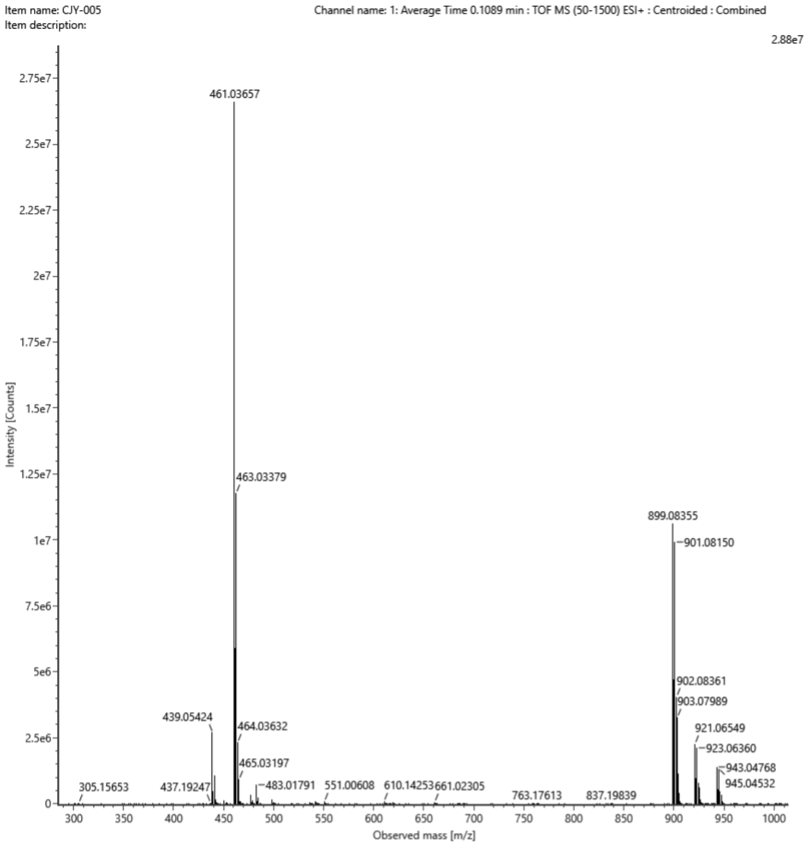

HRMS (ESI) of compound **4k**

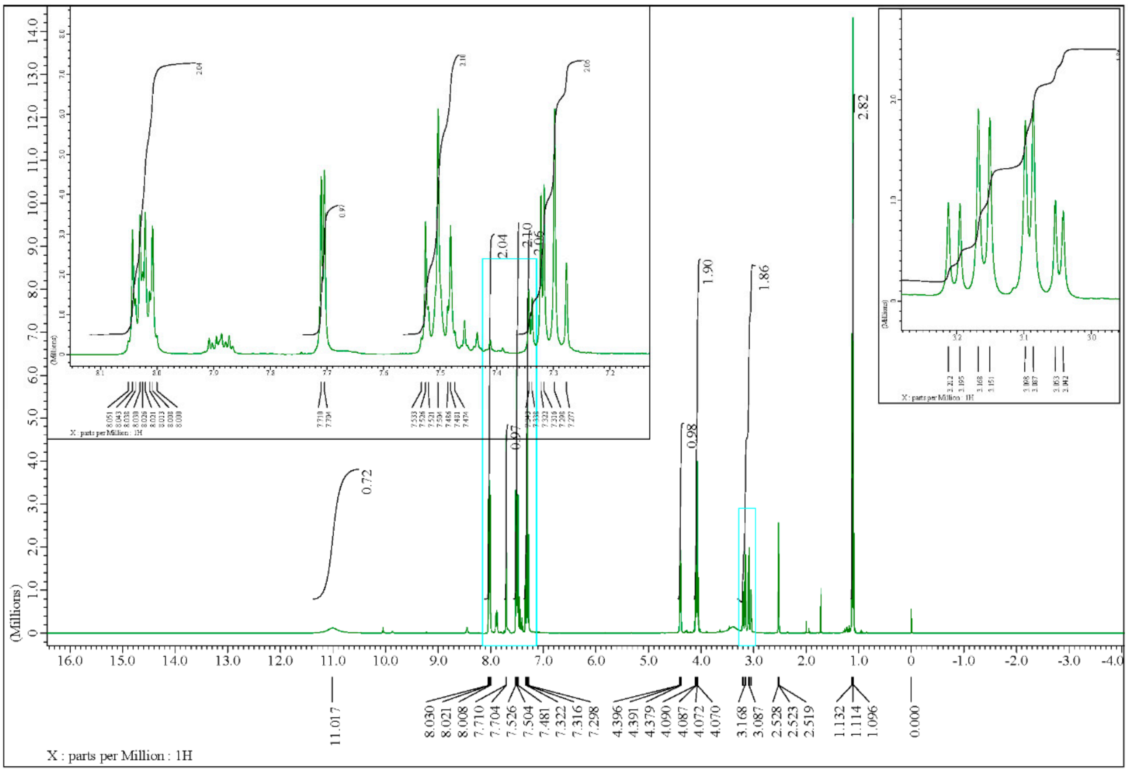

^{1}H NMR of compound **4l**

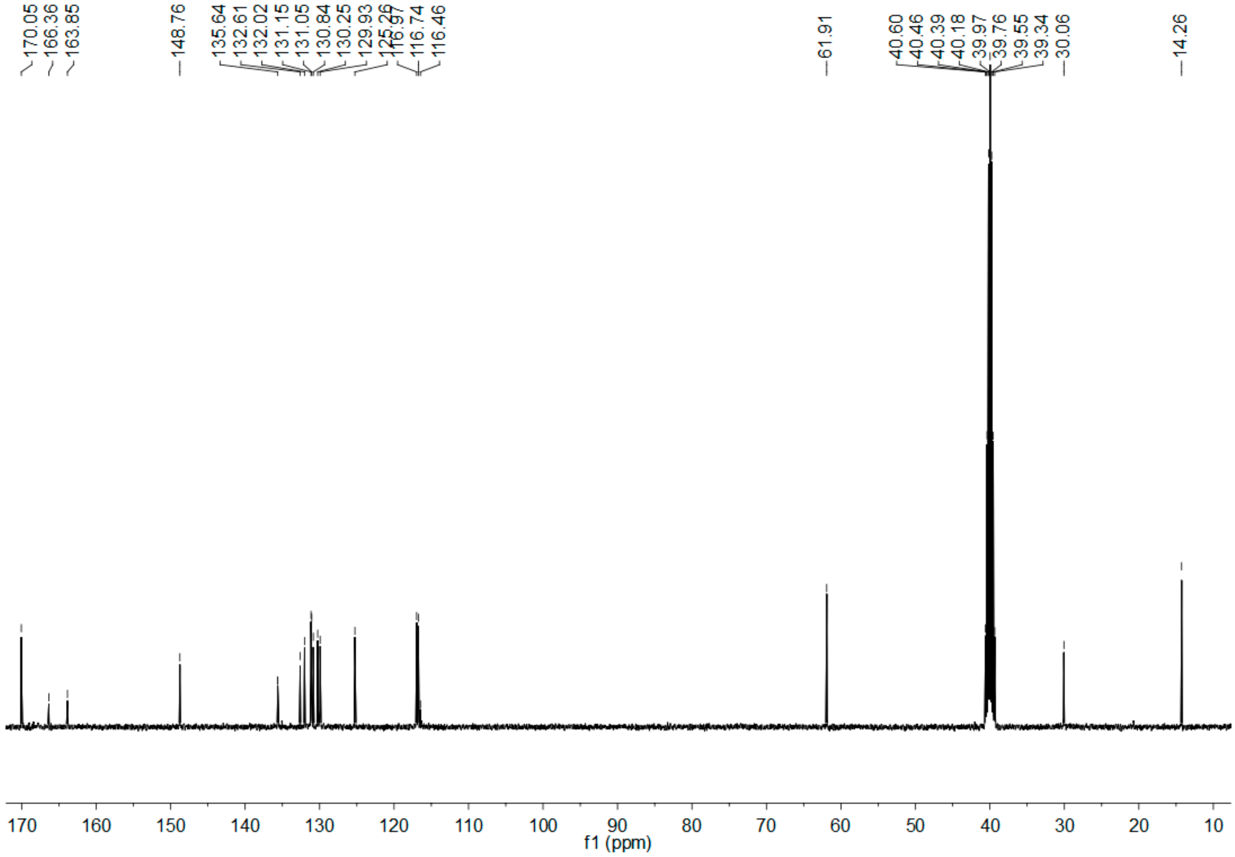

^{13}C NMR of compound **4l**

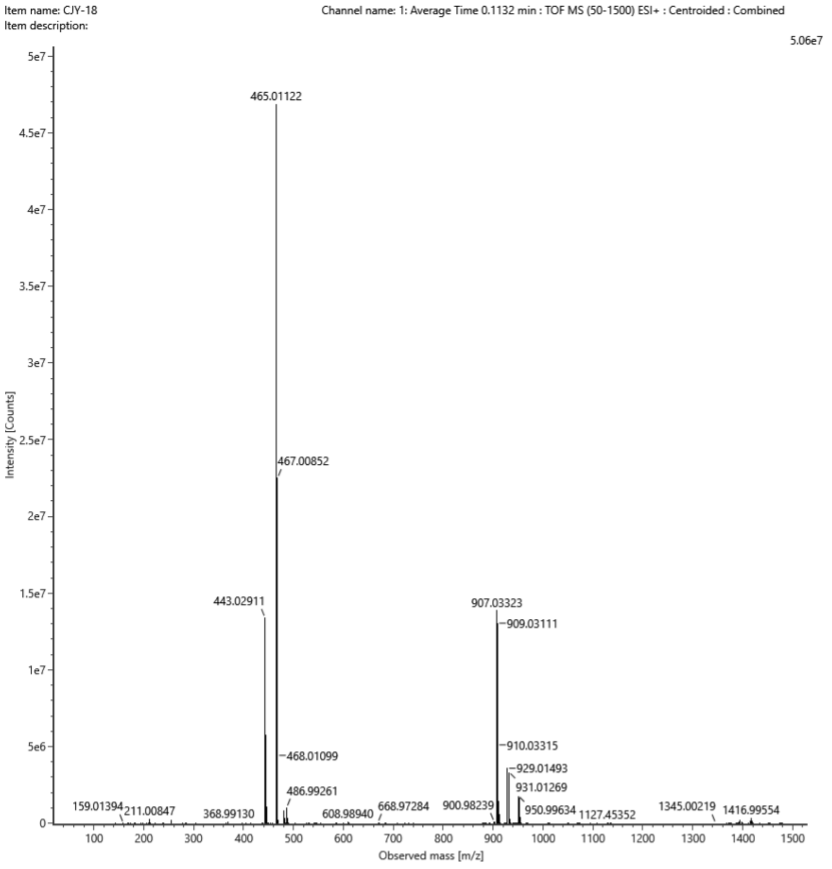

HRMS (ESI) of compound **4l**

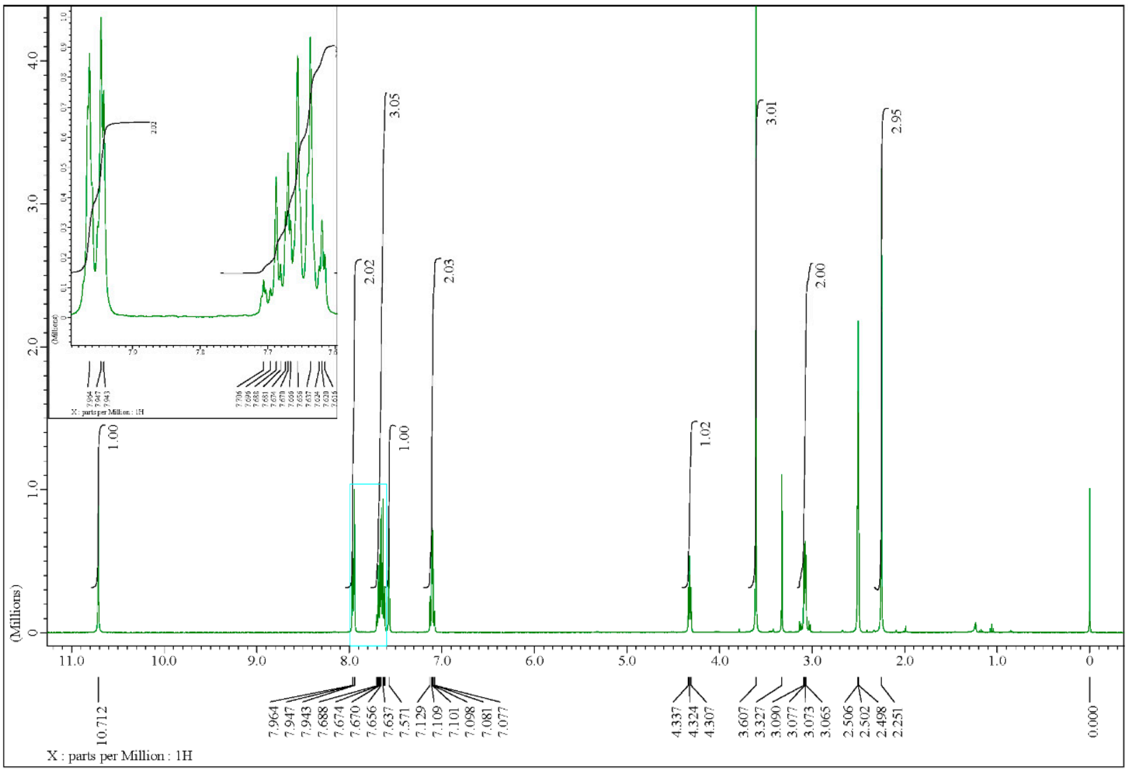

^{1}H NMR of compound **4m**

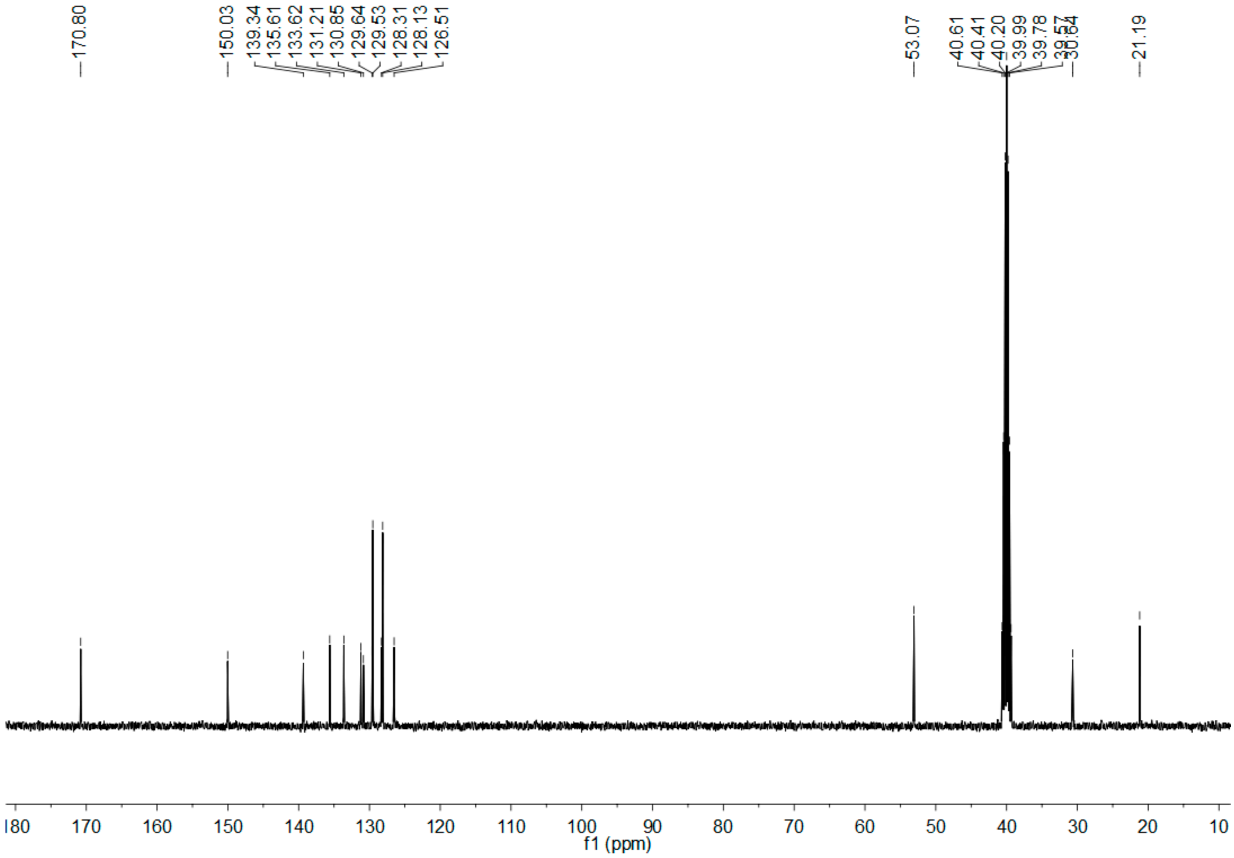

^{13}C NMR of compound **4m**

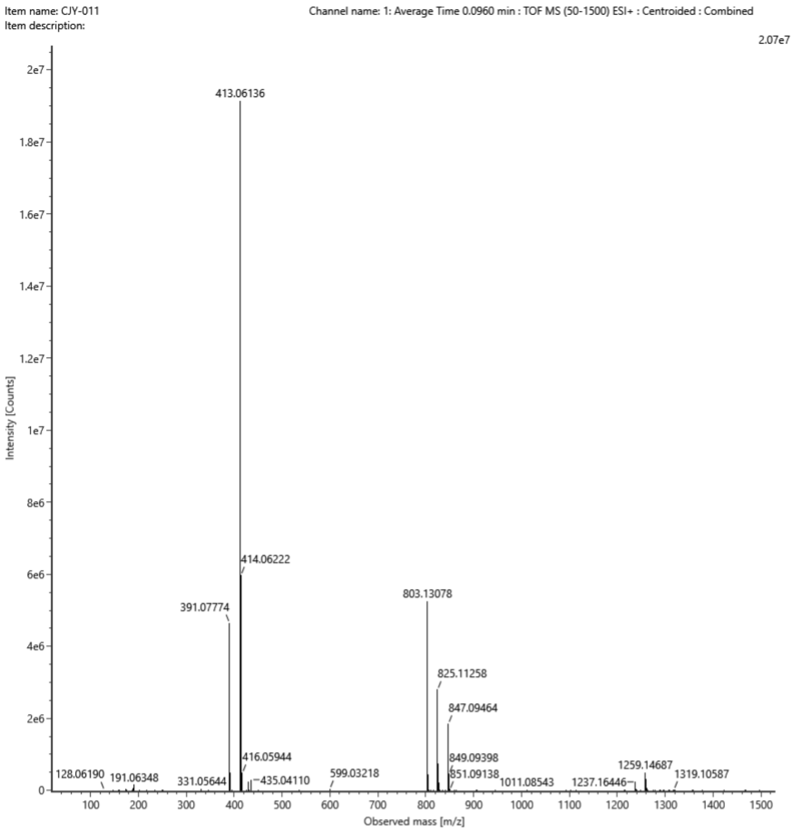

HRMS (ESI) of compound **4m**

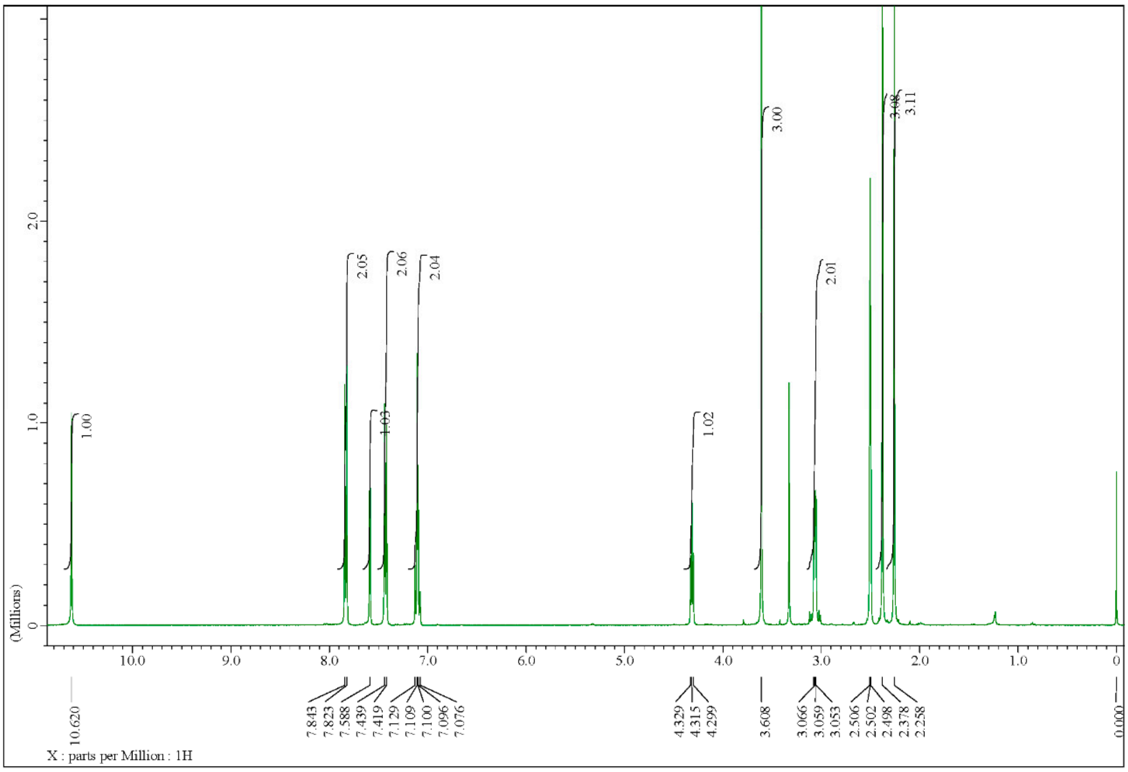

^{1}H NMR of compound **4n**

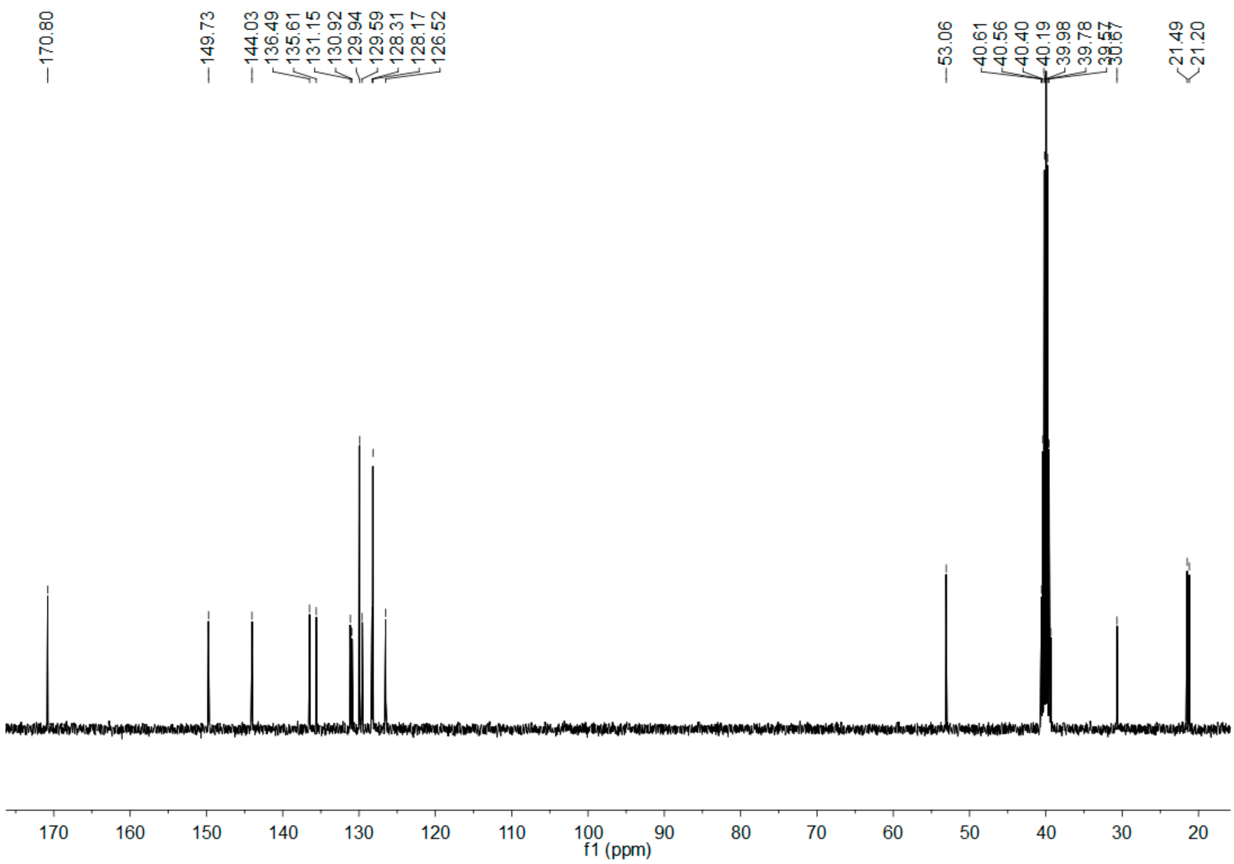

^{13}C NMR of compound **4n**

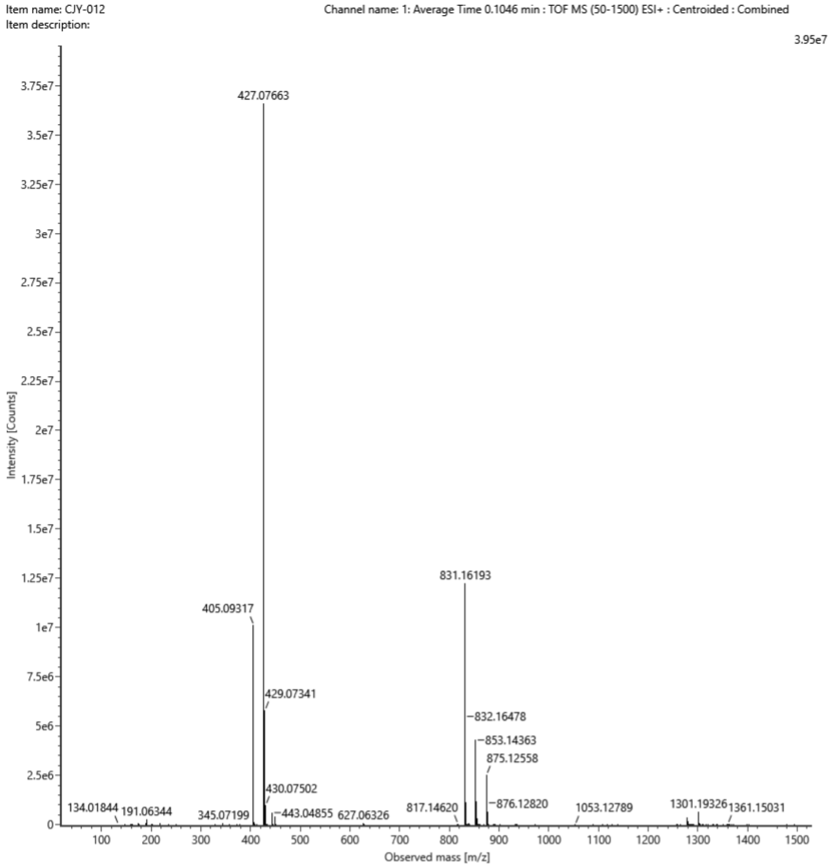

HRMS (ESI) of compound **4n**

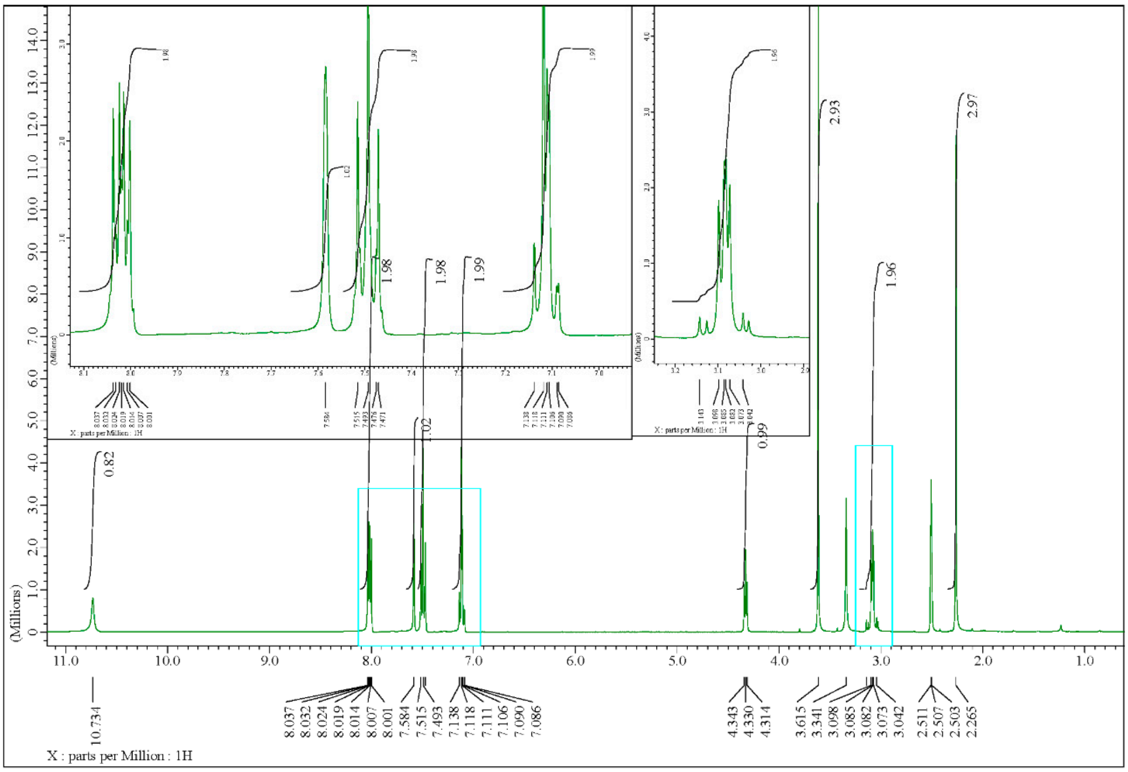

^{1}H NMR of compound **4o**

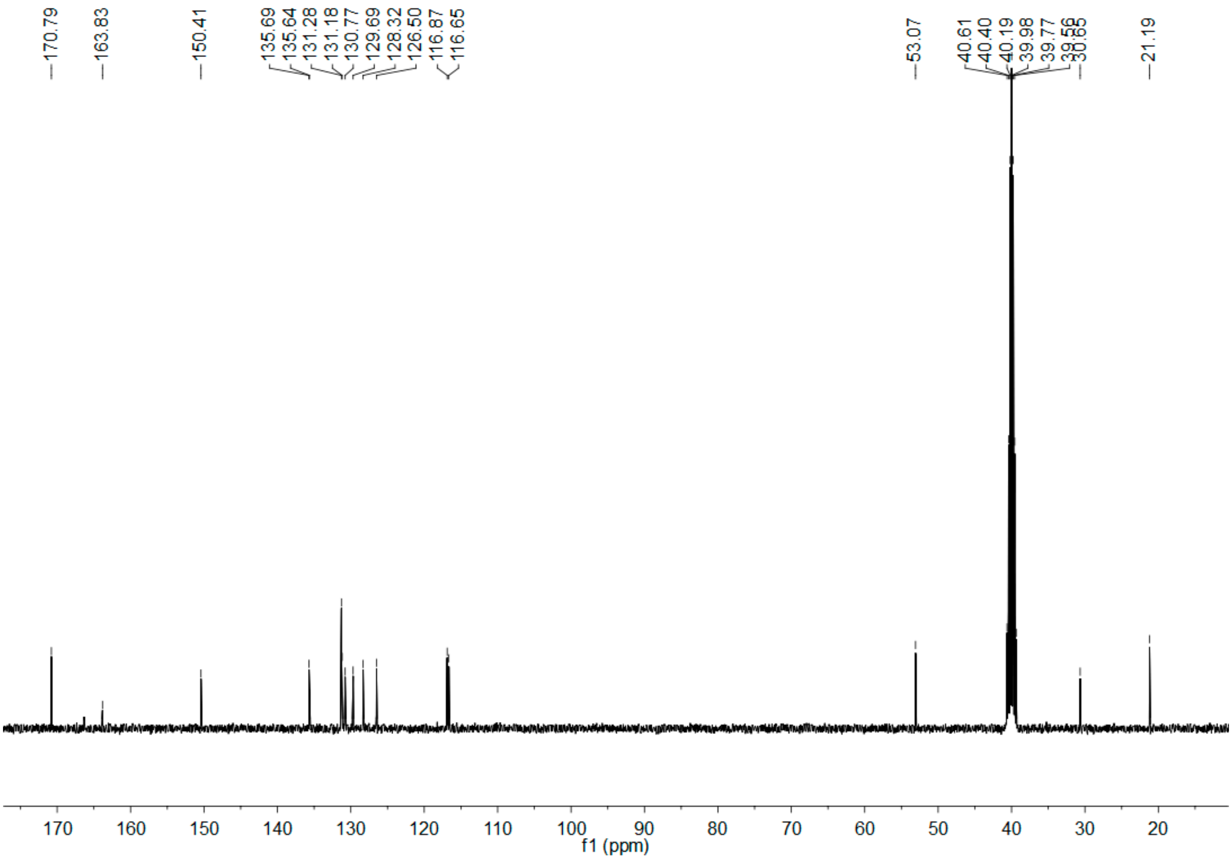

^{13}C NMR of compound **4o**

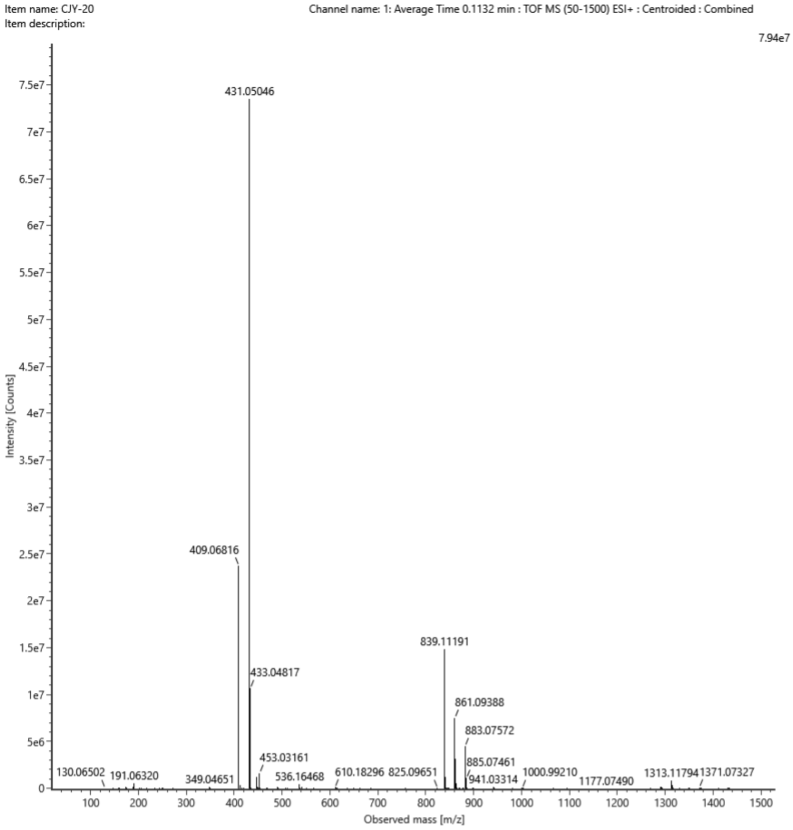

HRMS (ESI) of compound **4o**

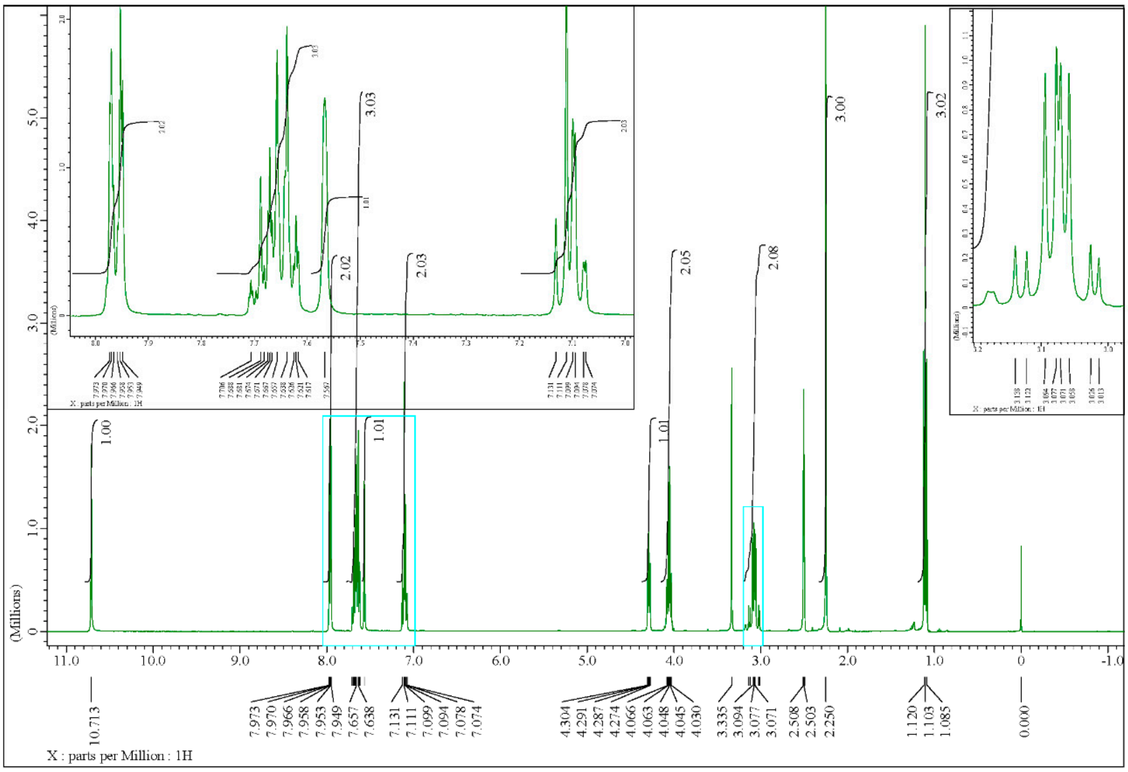

^{1}H NMR of compound **4p**

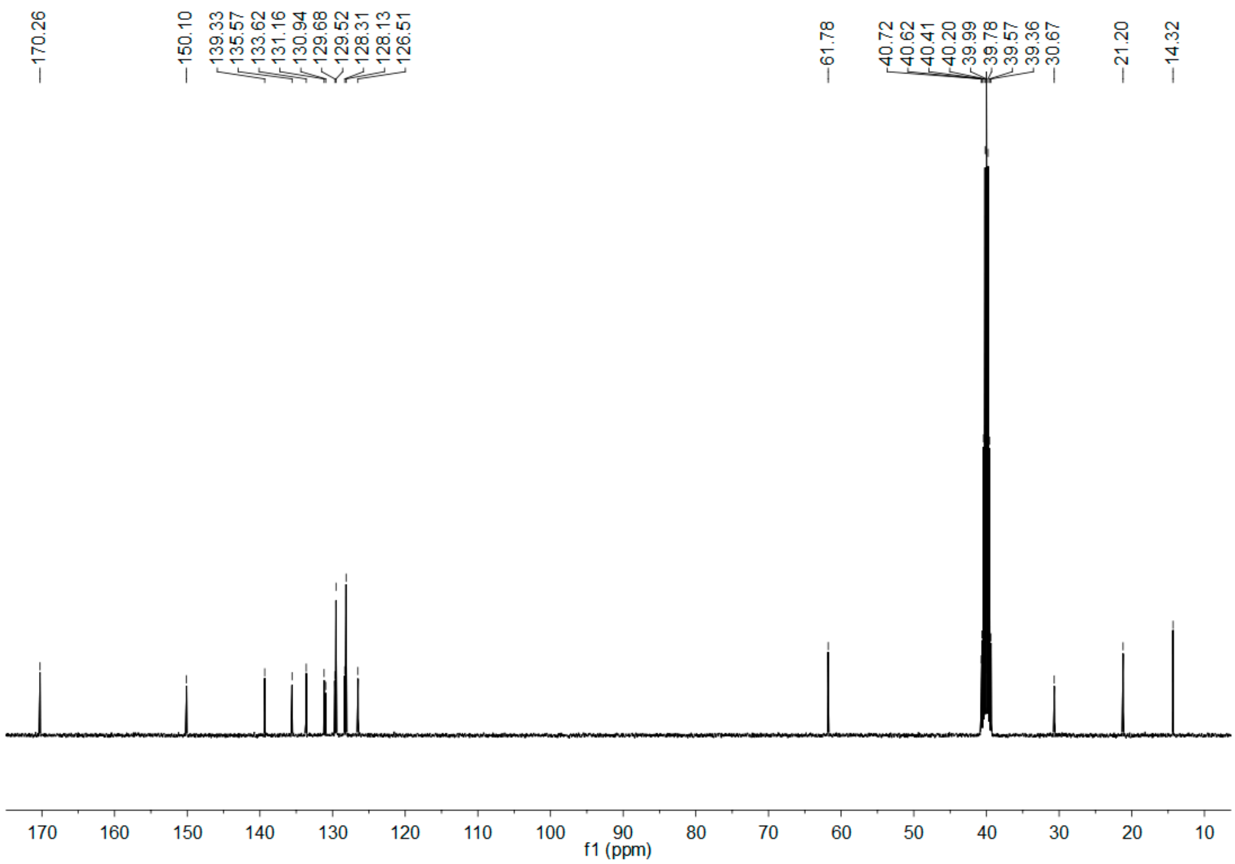

^{13}C NMR of compound **4p**

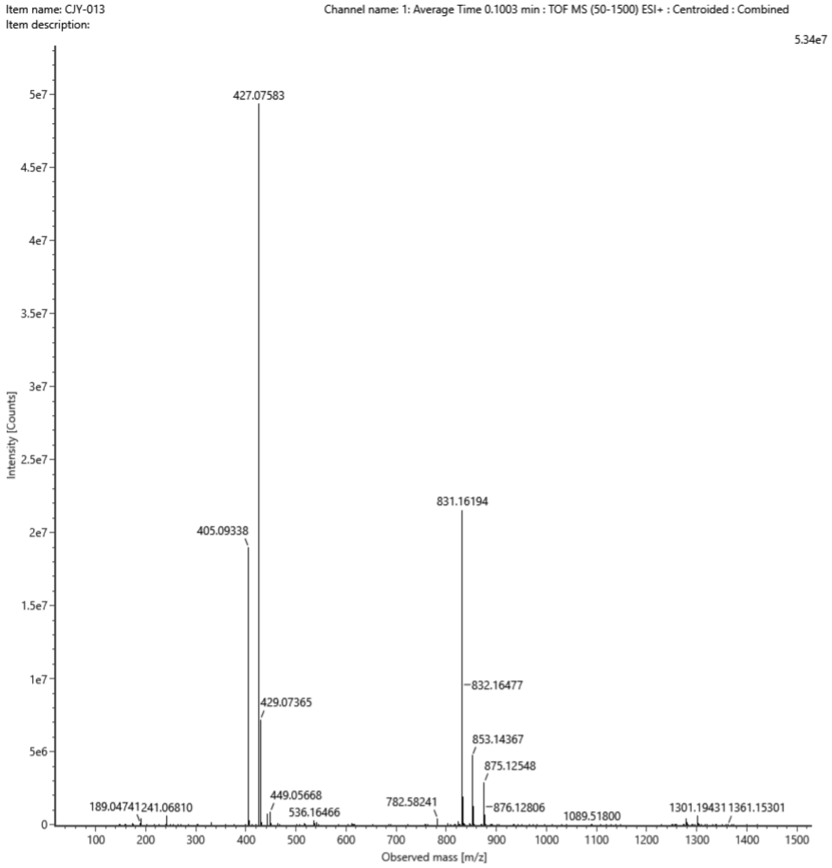

HRMS (ESI) of compound **4p**

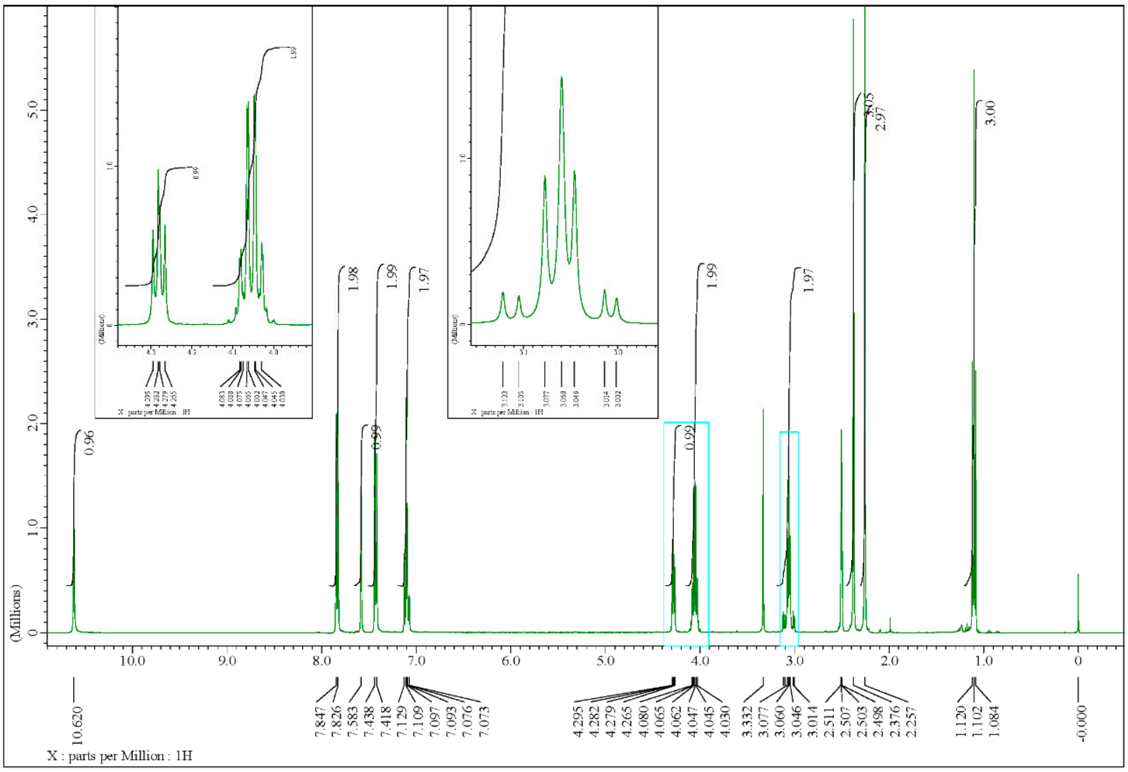

^{1}H NMR of compound **4q**

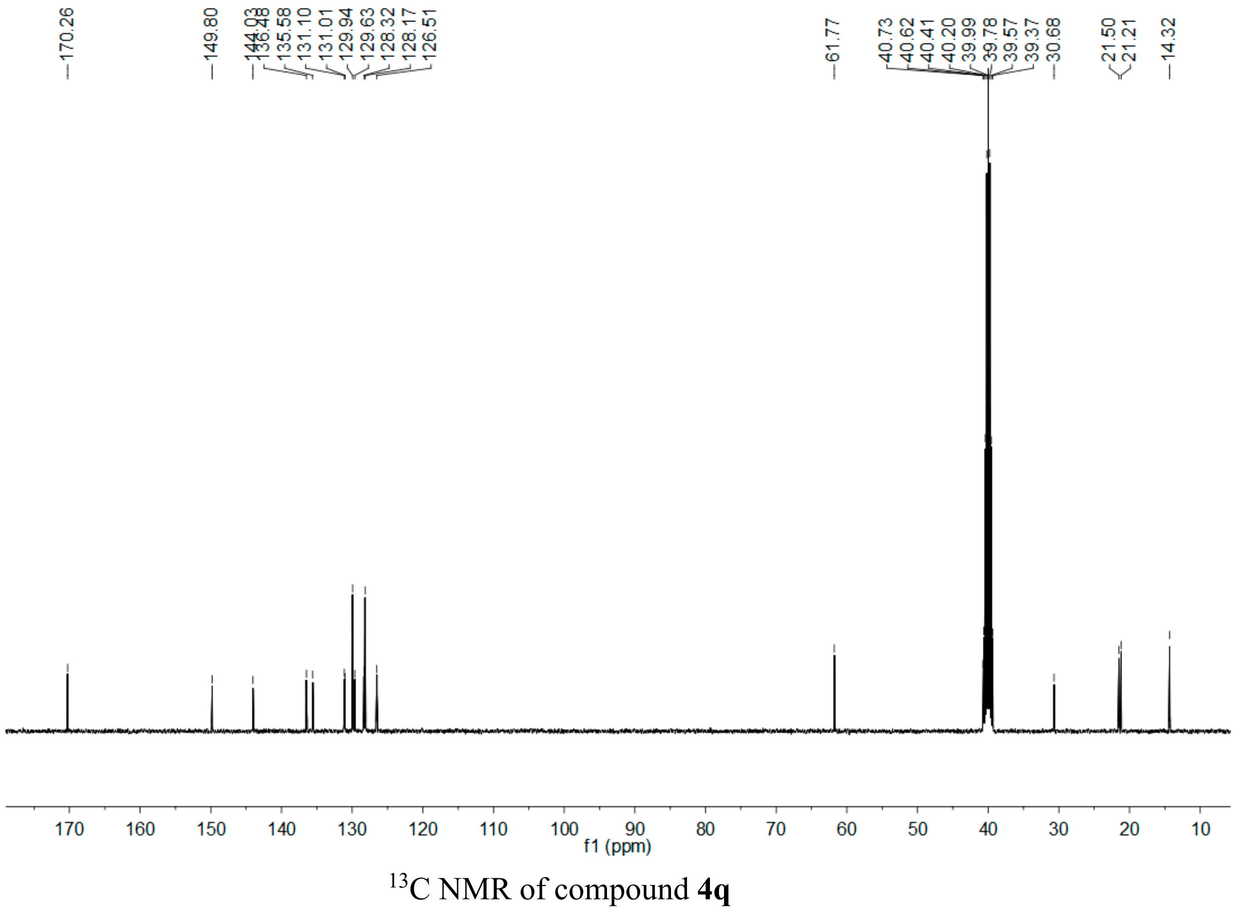

^{13}C NMR of compound **4q**

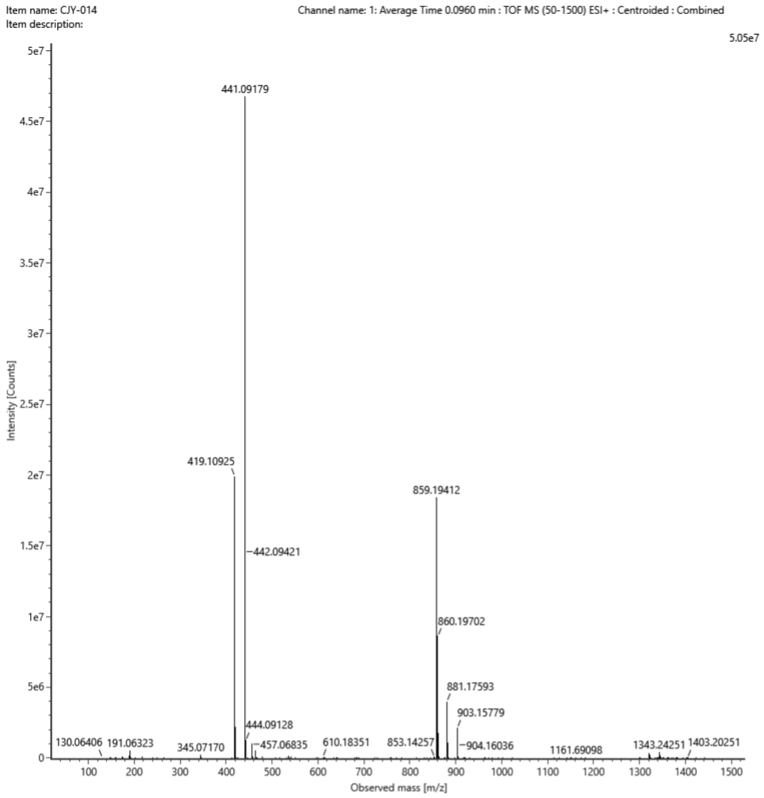

HRMS (ESI) of compound **4q**

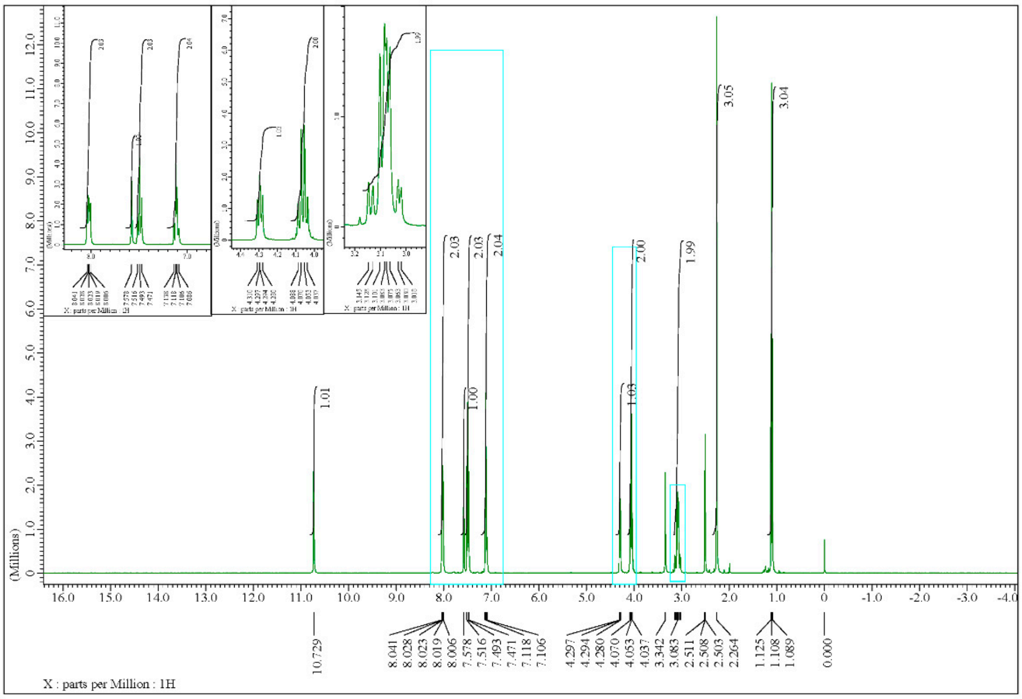

^{1}H NMR of compound **4r**

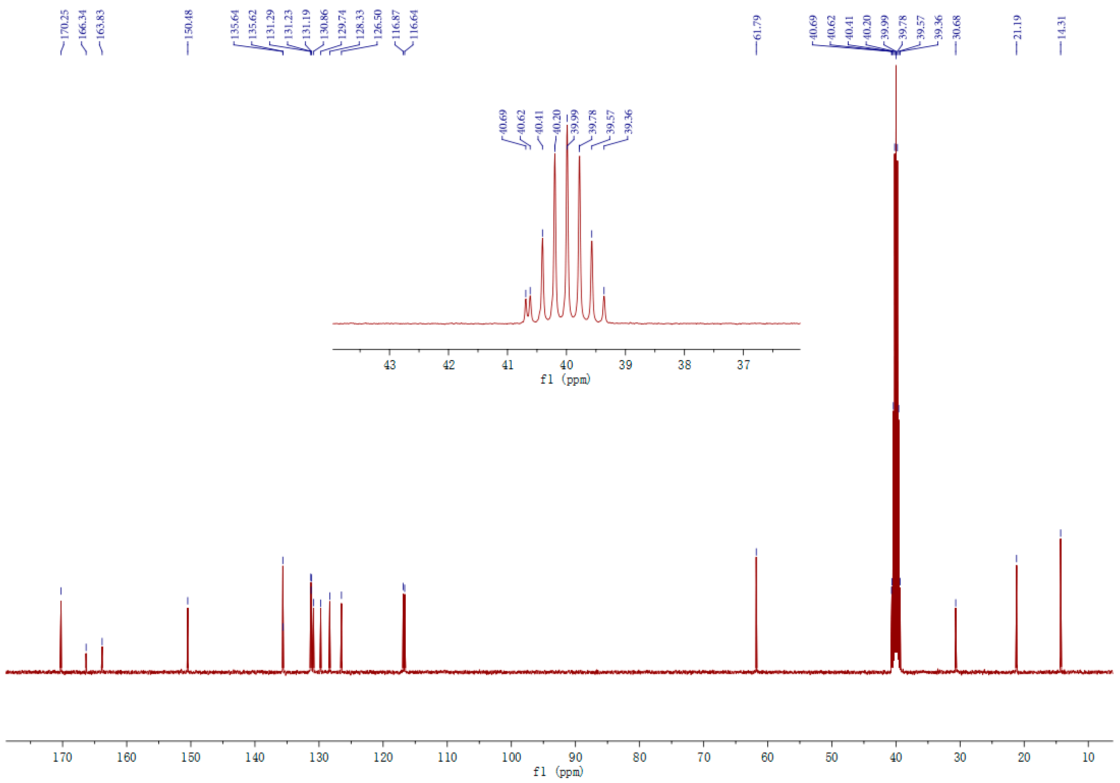

^{13}C NMR of compound **4r**

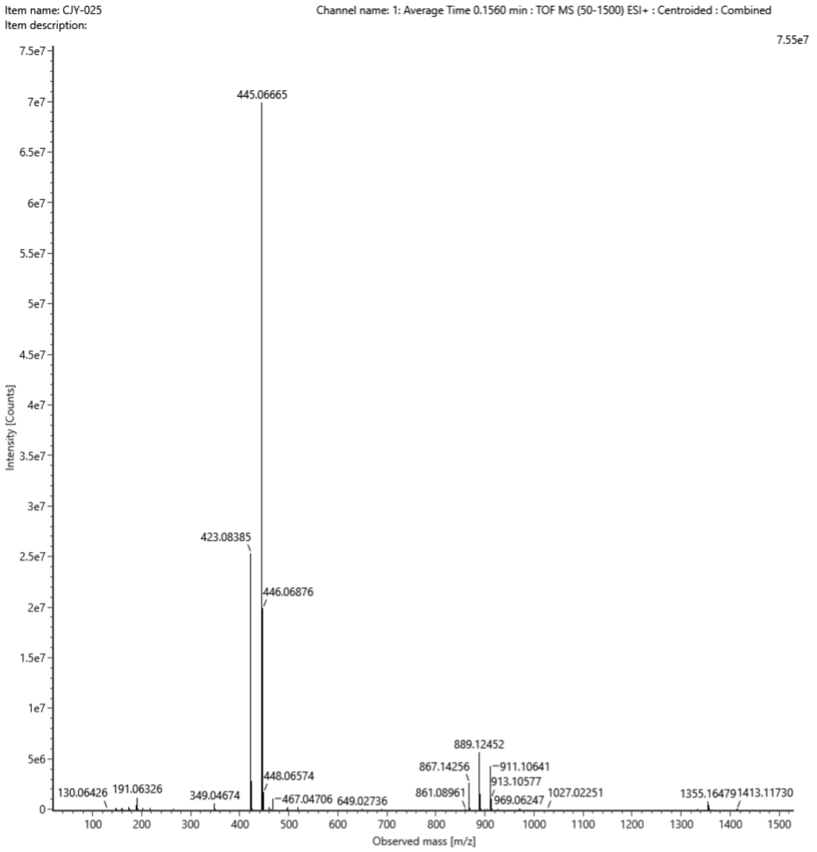

HRMS (ESI) of compound **4r**
